# Modeling between-population variation in COVID-19 dynamics in Hubei, Lombardy, and New York City

**DOI:** 10.1073/pnas.2010651117

**Published:** 2020-09-24

**Authors:** Bryan Wilder, Marie Charpignon, Jackson A. Killian, Han-Ching Ou, Aditya Mate, Shahin Jabbari, Andrew Perrault, Angel N. Desai, Milind Tambe, Maimuna S. Majumder

**Affiliations:** ^a^John A. Paulson School of Engineering and Applied Sciences, Harvard University, Cambridge, MA 02138;; ^b^Institute for Data, Systems, and Society, Massachusetts Institute of Technology, Cambridge, MA 02142;; ^c^Department of Internal Medicine, Division of Infectious Diseases, University of California-Davis Health, Sacramento, CA 95817;; ^d^Department of Pediatrics, Harvard Medical School, Boston, MA 02115;; ^e^Computational Health Informatics Program, Boston Children’s Hospital, Boston, MA 02115

**Keywords:** COVID-19, SARS-CoV-2, modeling, nonpharmaceutical intervention

## Abstract

As the COVID-19 pandemic continues, formulating targeted policy interventions that are informed by differential severe acute respiratory syndrome coronavirus 2 (SARS-CoV-2) transmission dynamics will be of vital importance to national and regional governments. We develop an individual-level model for SARS-CoV-2 transmission that accounts for location-dependent distributions of age, household structure, and comorbidities. We use these distributions together with age-stratified contact matrices to instantiate specific models for Hubei, China; Lombardy, Italy; and New York City, United States. Using data on reported deaths to obtain a posterior distribution over unknown parameters, we infer differences in the progression of the epidemic in the three locations. We also examine the role of transmission due to particular age groups on total infections and deaths. The effect of limiting contacts by a particular age group varies by location, indicating that strategies to reduce transmission should be tailored based on population-specific demography and social structure. These findings highlight the role of between-population variation in formulating policy interventions. Across the three populations, though, we find that targeted “salutary sheltering” by 50% of a single age group may substantially curtail transmission when combined with the adoption of physical distancing measures by the rest of the population.

## Significance

We present an individual-level model of severe acute respiratory syndrome coronavirus 2 transmission that accounts for population-specific factors such as age distributions, comorbidities, household structures, and contact patterns. The model reveals substantial variation across Hubei, Lombardy, and New York City in the dynamics and progression of the epidemic, including the consequences of transmission by particular age groups. Across locations, though, policies combining “salutary sheltering” by part of a particular age group with physical distancing by the rest of the population can mitigate the number of infections and subsequent deaths.

Since December 2019, the COVID-19 pandemic—propagated by the novel coronavirus severe acute respiratory syndrome coronavirus 2 (SARS-CoV-2)—has resulted in significant morbidity and mortality (1). As of 1 August 2020, an estimated 18 million individuals have been infected, with over 700,000 fatalities worldwide (2). Key factors such as existing comorbidities and age appear to play a role in an increased risk of mortality (3). Epidemiological studies have provided significant insights into the disease and its transmission dynamics to date (4–7). However, as national and regional governments begin to implement broad-reaching policies in response to rising case counts and stressed healthcare systems, tailoring these polices based on an understanding of how population-specific demography impacts outbreak dynamics will be vital. Previous modeling studies have not incorporated the rich set of household demographic features needed to address such questions.

**Fig. 1.**
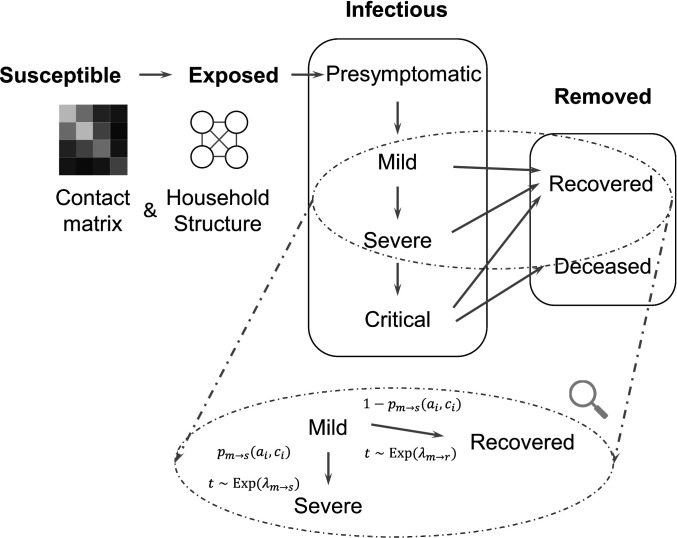
We use a modified SEIR model, where the infectious states are subdivided into levels of disease severity. The transitions are probabilistic and there is a time lag for transitioning between states. For example, the magnified section shows the details of transitions between mild, recovered, and severe states. Each arrow consists of the probability of transition [e.g., $p_{m\to s}\left(a_{i},c_{i}\right)$ denotes the probability of progressing from mild to severe] as well as the associated time lag (e.g., the time t for progression from mild to severe is drawn from an exponential distribution with mean $\lambda_{m\to s}$). $a_{i}$ and $c_{i}$ denote the age and set of comorbidities for the infected individual i.

This study develops a stochastic agent-based model for SARS-CoV-2 transmission which accounts for distributions of age, household types, comorbidities, and contact between different age groups in a given population (Fig. 1). Our model accounts for both within-household contact (simulated via household distributions taken from census data) and out-of-household contact using age-stratified, country-specific estimated contact matrices (8). We instantiate the model for Hubei, China; Lombardy, Italy; and New York City, United States, developing a Bayesian inference strategy for estimating the distribution of unknown parameters using data on reported deaths in each location. This enables us to uncover differences in the progression of the epidemic in each location. We also examine how transmission by particular age groups contributes to infections and deaths in each location, allowing us to compare the efficacy of efforts to reduce transmission across said groups. There is large between-population variation in the role played by any individual age group. However, across populations, both infections and deaths are substantially reduced by a combination of population-wide physical distancing and “salutary sheltering”—a term we coin here to describe individuals who shelter in place irrespective of their exposure or infectious state—by half the individuals in a specific age group, without the need for potentially untenable policies such as indefinite sheltering of all older adults.

## Results

### Inferring Differences in Dynamics between Populations.

Using our model, we estimate posterior distributions over unobserved quantities which characterize the dynamics of the epidemic in a particular location. This section presents estimates for two quantities: first, the basic reproduction number $r_{0}$, and second, the rate at which infections are documented. Neither quantity is directly observable in the data due to substantial underdocumentation of infections; however, these estimates are needed to characterize the scope of the outbreak in a particular location, the degree to which existing testing strategies capture new infections, and the rate at which infections are expected to increase in the absence of any intervention. These findings are critical to formulate policy interventions that are tailored to the outbreak as it evolves in a given population. We start by providing a brief overview of our inference strategy and model validation and then present the main estimates.

There are four model parameters for which values are not precisely estimated in the literature. Each such parameter is instead drawn from a prior distribution. First is $p_{\text{inf}}$, the probability of infection given contact with an infected individual. This determines the level of transmissibility of the disease. Second is $t_{0}$, the start time of the infection, which is not precisely characterized in most locations and has an impact due to rapid doubling times. Third is a parameter $d_{\text{mult}}$, which accounts for differences in mortality rates between locations that are not captured by demographic factors in the model (e.g., the impact of variation in health system capacities). $d_{\text{mult}}$ is a multiplier to the baseline mortality rate from ref. 9 and is applied uniformly across age groups. We also include an age-specific multiplier to the mortality rate for individuals over 60 y of age in Lombardy, which is calibrated independently of the other parameters to match the fraction of deaths attributed to the 60+-y age group [which is significantly higher in Lombardy than the other two locations (9–11)]. Further discussion of the age-specific distribution of deaths can be found in *SI Appendix*. Fourth is $\delta_{\text{c}}$, the reduction in person-to-person contact after mobility restrictions were imposed in each location. Following mobility restrictions, the expected number of contacts between agents in any two age groups outside the household is reduced to $\delta_{\text{c}}$ times its starting value. For Hubei, we fix this parameter using a post-lockdown contact survey (12). For Lombardy and New York City, post-lockdown surveys are not available and so we estimate $\delta_{\text{c}}$ within the Bayesian framework. Details of the prior distributions and the modeled scenario in each location can be found in *SI Appendix*.

**Fig. 2.**
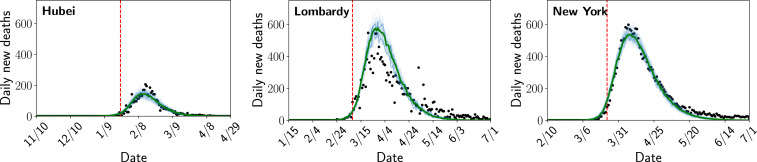
Posterior distribution over the number of deaths each day compared to the number of reported deaths. Light blue lines are individual samples from the posterior, green is the median, and the black dots are the number of reported deaths. The red dashed line represents the start of modeled contact reductions in each location.

By conditioning on the observed time series of deaths, we obtain a joint posterior distribution over both the unobserved model states, such as the number of people infected at each time step, as well as the three unknown parameters. We use reported deaths because they are believed to be better-documented than infections and perform a sensitivity analysis to account for possible underdocumentation of deaths (13, 14). Fig. 2 shows that the model closely reproduces the observed time series of deaths in each location. In *SI Appendix*, Figs. S1–S3 we also perform out-of-sample validation by fitting the model using a portion of the time series and assessing the accuracy of the predictive posterior distribution on data that was not used to fit the model.

**Fig. 3.**
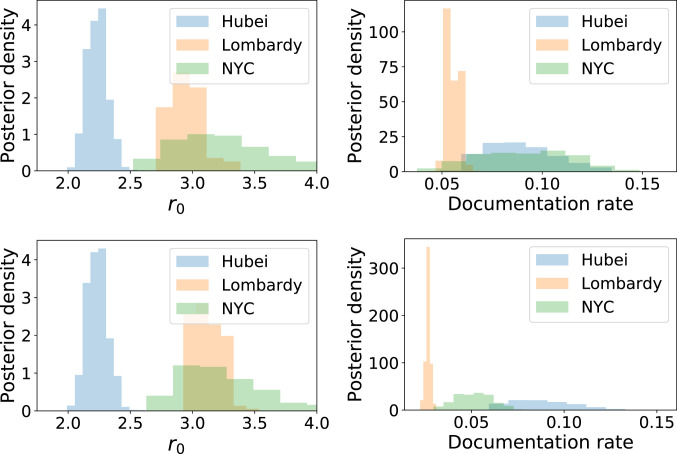
Posterior distribution over $r_{0}$ and the fraction of infections documented in each location (*Top*) conditioning on reported deaths and (*Bottom*) conditioning on deaths in New York City and Lombardy being twice what was reported.

Fig. 3, *Left* shows the posterior distribution over $r_{0}$ in each location. Substantial differences are evident between the three locations. The posterior median is 2.23 in Hubei (90% credible interval: 2.10 to 2.37), 2.95 in Lombardy (2.80 to 3.19), and 3.20 in New York City (2.71 to 3.93). The estimates for Hubei fall within the range of a number of existing estimates (15), while the interval for Lombardy is similar to the interval 2.9 to 3.2 estimated by previous work (16). The estimated $r_{0}$ for New York City is larger than either Hubei or Lombardy. The relative ranking of $r_{0}$ for the three populations is not impacted by a sensitivity analysis for underreporting of deaths, shown in Fig. 3. Death totals from Hubei have been substantially revised upward to correct for underreporting in the early stages of the epidemic (17), but such corrections are either unavailable or rapidly evolving for Lombardy and New York City. Our sensitivity analysis assumes that deaths in Lombardy and New York City are twice what was reported, consistent with preliminary investigations of excess mortality data (13, 14). In this scenario, the posterior median value of $r_{0}$ rises slightly to 3.12 in Lombardy and remains constant (at 3.20) in New York City. However, the estimated value of $\delta_{\text{c}}$ for each location rises sharply, indicating that the model explains increased deaths in this scenario via the possibility of less severe contact reductions during lockdown.

Fig. 3, *Right* shows the posterior distribution over the fraction of infections that were documented in each location (obtained by dividing the number of confirmed cases in each location by the number of infections in the simulation under each sample from the posterior). Documentation rates are uniformly low, indicating undocumented infections in all locations; however, we estimate lower documentation in Lombardy (90% credible interval: 5.1 to 6.0%) than in either New York City (5.4 to 12.7%) or Hubei (6.4 to 12.1%). Documentation rates are substantially lower when assuming twice the reported deaths in Lombardy and New York City (Fig. 3, *Bottom*).

Although we estimate a substantial number of undocumented infections, all locations remain potentially vulnerable to second-wave outbreaks, with the median percentage of the population infected at 1.3% in Hubei, 13.8% in Lombardy, and 22.0% in New York City. Note that in Hubei our estimate is for the entire province of Hubei, with a population of 58.5 million people, including—but not limited to—the city of Wuhan. Recent serological surveys have estimated 25% of the population previously infected in New York City (18), consistent with our distribution. When assuming that deaths are underreported by a factor of 2 in Lombardy and New York City, the median percentage infected is 28.2% in Lombardy and 38.7% in New York City* . Overall, our estimates for $r_{0}$ and the remaining population of susceptible individuals indicate that Hubei, Lombardy, and New York City could experience new outbreaks in the absence of continued interventions to reduce transmission. Despite this, between-population differences remain substantial; Hubei, Lombardy, and New York City have each had distinct experiences with COVID-19 that must be considered with respect to future policy responses.

### Containment Policies: Salutary Sheltering and Physical Distancing.

Various interventions—from complete lockdown to physical distancing recommendations—have been implemented worldwide in response to COVID-19. Within these are a range of alternatives. For example, a government could encourage some percentage of a given age group to remain sheltered in place, while the rest of the population could continue in-person work and social activities. Age-specific policies are particularly relevant because they have already been employed in some countries [e.g., US Centers for Disease Control and Prevention recommendations that people above 65 y old shelter in place (20)] and because older age groups are more likely to be able to telecommute, at least in the United States (21, 22).

Here, we investigate to what extent a second-wave outbreak in each of our three locations of interest can be mitigated by encouraging a single age group to engage in salutary sheltering or whether the entire population must also be asked to adopt physical distancing. We compare scenarios that combine varying levels of two different interventions: 1) salutary sheltering by a given fraction of a single age group modeled by eliminating all outside-of-household contact for agents who engage in sheltering and and 2) physical distancing by the population as a whole, modeled by reducing the expected number of outside-of-household contacts between all agents (who are not engaging in salutary sheltering) to a given percentage of their original value. While this case study applies to Hubei, Lombardy, and New York City, it could be extended to other locations using population-specific demographic data as well. *SI Appendix* includes details of all experiments described along with sensitivity analyses where the impact of physical distancing is further varied and where the population begins in a completely susceptible state (*SI Appendix*, Figs. S5–S8).

**Fig. 4.**
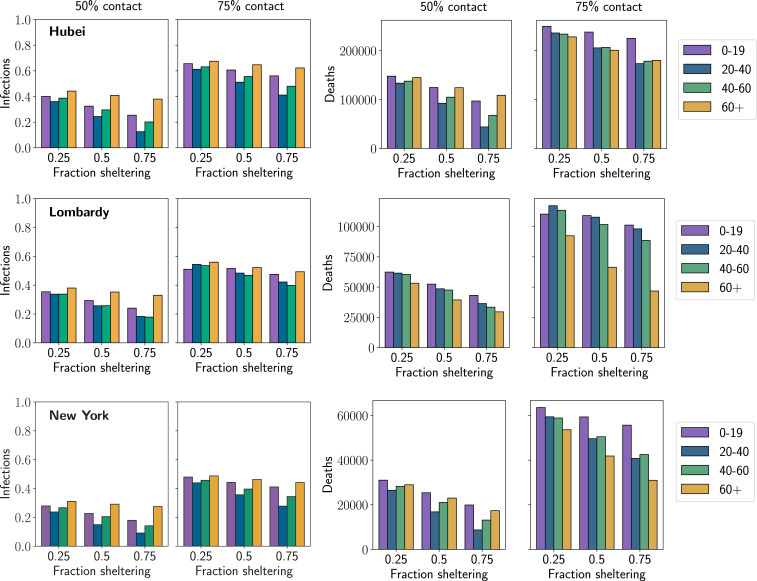
Number of new infections and new deaths in second-wave outbreak scenarios for each location. Each column shows a different level of physical distancing by the population as a whole, where contacts between all age groups are reduced to the given percentage of their starting value. The x axis within each plot shows the result when the given fraction of a single age group shelters at home (in addition to physical distancing by the rest of the population). The result of this combination of sheltering and distancing is represented by a bar, where the color of the bar indicates the age group which engaged in sheltering (see key). The height of the bar gives the total number of infections or deaths in the population in that scenario. Each row gives the results for a single location, where the first two plots show the fraction of the population which is newly infected in the second wave and the next two plots show the number of new deaths which occur.

Fig. 4 shows the number of new infections or deaths in each location during the second wave as we vary three quantities: 1) the reduction in contacts due to physical distancing by the entire population, 2) the age group which engages in salutary sheltering, and 3) the fraction of that age group which shelters in place. All results are averages over population-level parameters from the posterior distributions estimated in the previous section. We highlight several main results. *SI Appendix* provides a further breakdown of results from each scenario in terms of infections and deaths in those above and below 60 y of age (*SI Appendix*, Tables S3–S14).

First, the marginal impact of salutary sheltering by different age groups in limiting infections in the second-wave outbreak depends on the level of physical distancing adopted by the rest of the population. When physical distancing is high (25% of the original level of contact, shown in *SI Appendix*), the second-wave outbreak never infects a significant number of people because the effective reproduction number remains below 1. When physical distancing is not widely adopted (75% of the original level of contact), the outbreak reaches a significant fraction of the population no matter which group engages in sheltering (at least 30% of the population and often more becomes infected). However, in the middle scenario (50% of the original level of contact), the population is in a state where sheltering by members of a group with a large number of average contacts can significantly reduce the extent of total infections. Typically, members of the 20- to 40-y and 40- to 60-y age groups have more contacts than those in older or younger groups (8), so sheltering by both these groups can sharply reduce the fraction of the population infected in the second wave.

Second, the importance of sheltering by each age group in preventing deaths varies according to the level of physical distancing adopted by the rest of the population. When returning to a near-normal level of contact makes infection of a significant fraction of the population unavoidable (75% of normal contact), deaths are most appreciably reduced by sheltering the 60+ age group, since older individuals are at much higher risk of death after infection than those in younger age groups. However, in the intermediate scenario of 50% contact reduction, it may be more effective for members of younger age groups (20 to 40 y or 40 to 60 y) to engage in salutary sheltering. While these individuals are typically at lower risk of death than those in the 60+ group, they also have a significantly larger number of average daily contacts (8). By sheltering, they help shield older groups from infection more effectively than if an equivalent fraction of the older group engaged in sheltering themselves.

Third, the impact of sheltering by these groups across different scenarios is impacted by between-population differences. Each population has differences in contact patterns, the estimated probability of infection on contact ($p_{\text{inf}}$), the fraction who were infected in the initial outbreak (assuming short-term immunity against reinfection during the second outbreak), and the vulnerability of older individuals. For example, sheltering by the 60+ age group reduces deaths much more substantially in Lombardy than in either Hubei or New York City because Italian fatalities are concentrated more heavily in older groups, with 95% of reported deaths in the 60+ age group compared to 80% in Hubei and 74% in New York City (9–11). As a result, it is still slightly preferable in terms of averted deaths to shelter the 60+ group in Lombardy even in scenarios where there would be an advantage to sheltering by younger groups in other locations (50% contact levels). Another example is in Hubei, where the fraction of the population that is newly infected in the second wave is larger than in either Lombardy or New York City (despite a lower estimated $r_{0}$ in Hubei). This is because we estimate that a nonnegligible portion of Lombardy and New York City were both previously infected, while the population of Hubei province is still almost entirely susceptible (discussed in the previous section). The interplay of demographics, social structures, and the impact of the first outbreak create a range of between-population differences across scenarios.

Building on this analysis of Hubei, Lombardy, and New York City, our model suggests that hybrid policies that combine targeted salutary sheltering by one subpopulation and physical distancing by the rest can substantially mitigate infections and deaths due to a second-wave outbreak. However, the relative importance of sheltering by different age groups is strongly impacted by the extent to which physical distancing is adopted by the rest of the population and by a range of factors which can differ between populations. This suggests that demography and behavior in a particular place must be carefully considered while developing population-level interventions. Our analysis can be readily extended to other locations by parameterizing our model for a new population using existing demographic data and age-stratified contact patterns, allowing analysis of population-specific interventions.

## Discussion and Future Work

In this study, we developed a model of SARS-CoV-2 transmission that incorporates household structure, age distributions, comorbidities, and age-stratified contact patterns in Hubei, Lombardy, and New York City and created simulations using available demographic information from these three locations. Our findings suggest that in some locations substantial reductions in SARS-CoV-2 spread can be achieved by less drastic options short of population-wide sheltering in place. Instead, targeted salutary sheltering of specific age groups combined with adherence to physical distancing by the rest of the population may be sufficient to thwart a substantial fraction of infections and deaths. Physical distancing could be achieved by engaging in activities such as staggered work schedules, increasing spacing in restaurants, and prescribing times to use the gym or grocery store. Specific mechanisms and considerations for implementing physical distancing are documented in *SI Appendix*. It is important to note that between-population differences in the impact of sheltering different age groups can be substantial. Contact patterns, household structures, and variation in fatality rates (whether due to demographics or factors such as health system capacity) all influence the number of infections or deaths averted by sheltering a particular group. Thus, the implementation of physical distancing and sheltering policies should be tailored to the dynamics of COVID-19 in a particular population.

From a pragmatic perspective, targeted salutary sheltering may not be realistic for all populations. Its feasibility relies on access to safe shelter, which does not reflect reality for all individuals. In addition, sociopolitical realities may render this recommendation more feasible in some populations than in others. Concerns for personal liberty, discrimination against subsegments of the population, and societal acceptability may prevent the adoption of targeted salutary sheltering in some regions of the world. Allowing salutary sheltering to operate on a voluntary basis using a shift system (rather than for indefinite time periods) may address some of these issues. Future work should formulate targeted recommendations about salutary sheltering and physical distancing by age group or other stratification adapted to a specific country’s workforce.

One strength of this study is our ability to assess targeted interventions such as salutary sheltering in a population-specific manner. Existing modeling work of COVID-19 has largely focused on simpler compartmental or branching process models which do not allow for such assessments. While these models have played an important role in estimating key parameters such as $r_{0}$ (5, 7) and the rate at which infections are documented (23), as well as in the evaluation of prospective nonpharmaceutical interventions (24, 25), they do not characterize how differences in demography impact the course of an epidemic in a particular location. Our focus on population-specific demography allows for further refinement of current mortality estimates and is a strength of this study. $r_{0}$ estimates in this study are generally comparable to other estimates in the literature (15), although our model yields higher estimates for New York City and Lombardy than Hubei—possibly due to differential mask-wearing practices (26) or adoption of behavioral interventions such as hand hygiene (27). Reporting rates estimated in this study were generally lower than those in prior studies (28), although the trend across locations is consistent. One potential explanation is that Russell et al. (28) estimate documentation from death data using a case fatality rate from the literature while our model uses an infection fatality rate (IFR). The IFR is lower because it includes all infections, not only those that become confirmed cases. A lower fatality rate in turn implies that each additional infection is less likely to result in death, and so a greater number of total infections are required to account for the observed number of deaths.

One key advantage of our framework is its flexibility. Our model is modifiable to test different policies or simulate additional features with greater fidelity across a variety of populations. Examples of future work that can be accommodated include analysis of contact tracing and testing policies, health system capacity, and multiple waves of infection after lifting physical distancing restrictions. Our model includes the necessary features to simulate these scenarios while remaining otherwise parsimonious, a desirable feature given uncertainties in data reporting.

This study is not without limitations, however. While several comorbidities associated with mortality in COVID-19 were accounted for, the availability of existing data limited the incorporation of all relevant comorbidities. Most notably, chronic pulmonary disease was not included although it has been associated with mortality in COVID-19 (29), nor was smoking, despite its prevalence in both China and Italy (30, 31). Gender-mediated differences were also excluded, which may be important for both behavioral reasons [e.g., adoption of hand washing (32, 33)] and biological reasons [e.g., the potential protective role of estrogen in SARS-CoV infections (34)]. Nevertheless, these factors can all be incorporated into the model as additional data become available.

Additionally, our second-wave scenarios assumed that individuals who were infected previously are immune to reinfection during the second wave. The duration of acquired immunity to SARS-CoV-2 has not been precisely defined, though antibody kinetics have been studied in recent work (35–37). If reinfection during a second wave is common, more individuals may be infected than predicted by our simulations (though mortality may be lower if previous infection is protective against adverse effects).

Finally, it is worth noting that we have not yet attempted to model super-spreader events in our existing framework. Such events may have been consequential in South Korea (38), and future work could attempt to model the epidemic there by incorporating a dispersion parameter into the contact distribution, a method which has been employed in other models (5).

Despite these limitations, this study demonstrates the importance of considering population and household demographics when attempting to better define outbreak dynamics for COVID-19. Furthermore, this model highlights potential policy implications for nonpharmaceutical interventions that account for population-specific demographic features and may provide alternative strategies for national and regional governments moving forward.

## Materials and Methods

This section provides an overview of our modeling and inference strategy. Additional details can be found in *SI Appendix*.

### Model.

We develop an agent-based model for COVID-19 spread which accounts for the distributions of age, household types, comorbidities, and contact between different age groups in a given population. The model follows a susceptible–exposed–infectious–removed (SEIR) template (39, 40). Specifically, we simulate a population of n agents (or individuals), each with an age $a_{i}$, a set of comorbidities $c_{i}$, and a household (a set of other agents). We stratify age into 10-y intervals and incorporate hypertension and diabetes as comorbidities due to their worldwide prevalence (41) and association with higher risk of in-hospital death for COVID-19 patients (3). However, our model can be expanded to include other comorbidities of interest in the future. The specific procedure we use to sample agents from the joint distribution of age, household structures, and comorbidities can be found in *SI Appendix*. We focus on modeling household contacts in particular detail because of the documented frequency of within-household transmission (7) and the previous suggestion that patterns of contact within the household may play a large role in shaping the epidemic (42). It is important to acknowledge that available data sources only suffice to model the joint distribution of age and household structure, whereas sampled comorbidities are conditioned only on the age of each agent (ignoring potential correlations between the comorbidity statuses of household members). However, this procedure still captures the marginal distribution of comorbidities over age in the population and hence the aggregate impact of COVID-19 on said population.

The disease is transmitted over a contact structure, which is divided into in-household and out-of-household groups. Each agent has a household consisting of a set of other agents (see *SI Appendix* for details on how households are generated using country-specific census information). Individuals infect members of their households at a higher rate than out-of-household agents. We model out-of-household transmission using country-specific estimated contact matrices (8). These matrices state the mean number of daily contacts an individual of a particular age stratum has with individuals from each of the other age strata. We assume demographics and contact patterns in each location are well-approximated by country-level data.

The model iterates over a series of discrete time steps, each representing a single day, from a starting time $t_{0}$ to an end time T. There are two main components to each time step: disease progression and new infections. The progression component is modeled by drawing two random variables for each individual each time they change severity levels (e.g., on entering the mild state). The first random variable is Bernoulli and indicates whether the individual will recover or progress to the next severity level. The second variable represents the amount of time until progression to the next severity level. We use exponential distributions for almost all time-to-event distributions, a common choice in the absence of specific distributional information (43, 44). The exception is the incubation time between presymptomatic and mild states, where more specific information is available; here, we use a log-normal distribution based on estimates in ref. 45. *SI Appendix*, Table S1 summarizes all distributions and their parameters and describes how we estimate age- and comorbidity-dependent severity progression. The “mild” state in our model encompasses the entire gradient of individuals who may have specific symptoms of COVID-19 but do not warrant hospitalization, those with paucisymptomatic or subclinical infections, and those with no detectable symptoms at all. Our model does not currently distinguish between the transmissibility of individuals in any of these states, which is not yet precisely characterized; however, it can be extended as more information becomes available.

In the new infections component, infected individuals infect each of their household members with probability $p_{h}$ at each time step. $p_{h}$ is calibrated so that the total probability of infecting a household member before either isolation or recovery matches the estimated secondary attack rate for household members of COVID-19 patients (i.e., the average fraction of household members infected) (46). Infected individuals draw outside-of-household contacts from the general population using the country-specific contact matrix. For an infected individual of age group i, we sample $w_{ij}^{s}∼\text{Poisson}\left(M_{ij}^{s}\right)$ contacts for each age group j and setting s where $M^{s}$ is the country-specific contact matrix for setting s. We include contacts in work, school, and community settings. Poisson distributions are a standard choice for modeling contact distributions (8). Then, we sample $w_{ij}^{s}$ contacts of age j uniformly with replacement, and each contact is infected with the probability $p_{\text{inf}}$, the probability of infection given contact. There is evidence to suggest that the probability of infection is higher for an older individual than a younger one given the same exposure (12), consistent with decline in immune function with age. We adjust for this by letting the probability of infection be $\beta p_{\text{inf}}$ when the exposed individual is over the age of 60 y, for β>1. β is calibrated to match the fraction of deaths in China attributed to individuals over the age of 60 y, resulting in a value of 1.25. This is consistent with the relationship between age and attack rate among close contacts of a confirmed case reported by (12), where the increase in risk of infection for a contact over 65 y old was estimated in the range 1.12 to 1.92.

### Inference of Posterior Distributions.

We infer unknown model parameters and states in a Bayesian framework. This entails placing a prior distribution over the unknown parameters and then specifying a likelihood function for the observable data, the time series of deaths reported in a location. We posit the following generative model for the observed deaths:

$$\begin{array}{ll} & p_{\text{inf}},d_{\text{mult}},t_{0}∼U\\ & d_{1}\ldots .d_{T}∼ABM\left(p_{\text{inf}},d_{\text{mult}},t_{0}\right)\\ & o_{t}∼\text{NegativeBinomial}\left(d_{t},\sigma_{obs}^{2}\right)\;t=1\ldots T, \end{array}$$

where U denotes a joint uniform prior, ABM denotes a draw from the stochastic agent-based dynamics, $d_{1}\ldots d_{T}$ are the time series output by the simulation, and $o_{1}\ldots o_{T}$ are the number of deaths observed on the corresponding dates. We model the observations as drawn from a negative binomial distribution (appropriate for overdispersed count data) with dispersion parameter $\sigma_{obs}^{2}$. We separately estimated $\sigma_{obs}^{2}$ by fitting an autoregressive negative binomial regression to the observed counts using the R package tscount (47). The negative binomial observation model was strongly preferred to a Poisson model (see *SI Appendix*, Table S2 with Akaike information criterion values). Together, the likelihood function is given by

$$L\left(p_{\text{inf}},d_{\text{mult}},t_{0},d_{1}\ldots d_{T}\right)=\overset{T}{\underset{t=1}{\prod }}\text{Pr}\left[o_{t}|d_{t},\sigma_{obs}^{2}\right].$$

To obtain the posterior distribution, we use Latin hypercube sampling to draw many (10,000 to 80,000 per location, depending on the size of the prior ranges) samples from the joint uniform prior over $p_{\text{inf}},d_{\text{mult}}$ and $t_{0}$ and then sample the latent variables $d_{1}\ldots d_{T}$ at each combination of parameters. We compute the likelihood for the full sample (including the latent variables). This allows us to use importance sampling to resample values of $\left(p_{\text{inf}},d_{\text{mult}},t_{0},d_{1}\ldots d_{T}\right)$ according to the posterior distribution. Finally, we marginalize out $d_{1}\ldots d_{T}$ to obtain the posterior over the parameters $p_{\text{inf}},d_{\text{mult}},t_{0}$, along with unobservable state variables of the simulation such as the number of infected individuals at each step.

## Supplementary Material

Supplementary File

## Data Availability

Code and data have been deposited in GitHub (https://github.com/bwilder0/covid_abm_release).

## Footnotes

*Of note, even in a scenario with substantially more deaths than documented, it is possible for the fraction infected to be lower than these estimates. Our model’s contact patterns capture the general population, but there is the potential for excess deaths to occur disproportionately in high-risk settings with anomalous contact patterns [e.g., reports have linked a large number of deaths to elder care facilities (19)]. In such circumstances, higher total deaths would not necessarily indicate a substantial increase in the fraction of the entire population infected.
