# Supplementary material for: Modeling between-population variation in COVID-19 dynamics in Hubei, Lombardy, and New York City
Source: Proc Natl Acad Sci U S A. 2020 Sep 24;117(41):25904–10. doi: 10.1073/pnas.2010651117 (PMC7568285; doi:10.1073/pnas.2010651117)
Supplement: Supplementary File [file pnas.2010651117.sapp.pdf]

PNAS
www.pnas.org

Supplementary Information for

Modeling between-population variation in COVID-19 dynamics in Hubei, Lombardy, and New York City

Bryan Wilder, Marie Charpignon, Jackson A. Killian, Han-Ching Ou, Aditya Mate, Shahin Jabbari, Andrew Perrault, Angel N. Desai, Milind Tambe, Maimuna S. Majumder

E-mail: bwilder@g.harvard.edu (B.W.); milind_tambe@harvard.edu (M.T.); Maimuna.Majumder@childrens.harvard.edu (M.S.M.)

This PDF file includes:

Supplementary text
Figs. S1 to S8
Tables S1 to S14
SI References

www.pnas.org/cgi/doi/10.1073/pnas.2010651117

Supporting Information Text

This section contains details of the methods (model used, parameter settings), strategy for inference, experimental details of the modeled scenarios, discussion of mechanisms for physical distancing, and additional results on model validation.

Methods

Model description. We develop an agent-based model for COVID-19 spread which accounts for the distributions of age, household types, comorbidities, and contact between different age groups in a given population. The model follows a *susceptible-exposed-infectious-removed (SEIR)* template (1, 2).

Specifically, we simulate a population of n agents (or individuals), each with an age a_i, a set of comorbidities c_i, and a household (a set of other agents). We stratify age into ten-year intervals and incorporate hypertension and diabetes as comorbidities. These comorbidities are common worldwide (3) and have been associated with a higher risk of in-hospital death for COVID-19 patients (4). However, our model can be expanded to include other comorbidities of interest in the future. The specific procedure we use to sample agents from the joint distribution of age, household structures, and comorbidities is described below.

The simulation tracks two states for each individual: the *infection state* and the *isolation state*. The infection state is divided into {*susceptible, exposed, infectious, removed*}. *Susceptible* individuals are those who have never been contacted by an infectious individual. *Exposed* individuals are those who have had contact with an infectious individual, though not all exposed individuals become infectious. If an exposed individual contracts the disease, they proceed to the infectious state.* *Infectious* is further subdivided into severity levels {*presymptomatic, mild, severe, critical*}. We interpret mild severity as symptomatic (but not requiring hospitalization), severe as requiring hospitalization, and critical as eligible for intensive care unit (ICU) care. The *removed* state is further subdivided into {*recovered*, *deceased*}. Individuals in all severity levels can transmit the disease, but those in the *presymptomatic* state do so at a rate $\alpha < 1$ times that of symptomatic cases. The decision to incorporate reduced transmission for presymptomatic individuals is based on the fact that, though infection by presymptomatic individuals has been observed in case clusters and in examinations of serial intervals (5–7), available evidence suggests that individuals with no or limited symptoms are less infectious than those with severe symptoms (8). Currently, our simulation incorporates two levels of infectiousness (before and after the onset of symptoms), but it can be adjusted as better information on how viral shedding increases with severity of illness becomes available. We acknowledge that our assumptions surrounding transmissibility and disease severity – as derived from existing literature – may serve as a limitation of our model, as many of these factors are evolving over time.

Each individual has a separate isolation state {*isolated*, *not isolated*}. If isolated, the individual is unable to infect others. We assume that (1) presymptomatic individuals are never isolated, (2) mild individuals become isolated over a mean time of λ_{isolate} days (see Table 1) after the onset of symptoms, and (3) all severe and critical individuals are isolated. However, our simulation framework can easily accommodate different sets of assumptions about isolation (for example, preemptively isolating exposed individuals if they are known to have had contact with an infectious agent).

The disease is transmitted over a contact structure, which is divided into in-household and out-of-household groups. Each agent has a household consisting of a set of other agents. Individuals infect members of their households at a higher rate than out-of-household agents. We model out-of-household transmission using country-specific estimated contact matrices (9). These matrices state the mean number of daily contacts an individual of a particular age strata has with individuals from each of the other age strata. We assume demographics (including age and household distribution) in Hubei and Lombardy are well-approximated by country-level data.

The model iterates over a series of discrete time steps, each representing a single day, from a starting time t_0 to an end time T. There are two main components to each time step: disease progression and new infections. The progression component is modeled by drawing two random variables for each individual each time they change severity levels (e.g., on entering the mild state). The first random variable is Bernoulli and indicates whether the individual will recover or progress to the next severity level. The second variable represents the amount of time until progression to the next severity level. We use exponential distributions for almost all time-to-event distributions, a common choice in the absence of specific distributional information (10, 11). The exception is the incubation time between presymptomatic and mild states, where more specific information is available; here, we use a log-normal distribution (see $\mu_{e\to m}$ and $\sigma^2_{e\to m}$ in Table 1) based on estimates by (12). Table 1 summarizes all distributions and their parameters.

In the new infections component, individuals in the susceptible state may enter the exposed state. Infected individuals infect each of their household members with probability p_h at each time step. p_h is calibrated so that the total probability of infecting a household member before either isolation or recovery matches the estimated secondary attack rate for household members of COVID-19 patients (i.e., the average fraction of household members infected) (13). Infected individuals draw outside-of-household contacts from the general population using the country-specific contact matrix. For an infected individual of age group i, we sample $w^s_{ij} \sim \text{Poisson}(M^s_{ij})$ contacts for each age group j and setting s where M^s is the country-specific contact matrix for setting s. We include contacts in work, school, and community settings. Poisson distributions are a standard choice for modeling contact distributions (9). Then, we sample w^s_{ij} contacts of age j uniformly with replacement, and each contact is infected with the probability p_{inf}, the probability of infection given contact. There is evidence to suggest that the

*Currently, our simulation implementation does not separately track individuals who are exposed but do not become infected, and instead groups them with the susceptible population. This is because we assume that, if exposed again, they will become infected with the same probability as an individual who has never been exposed. However, the implementation can be modified to support either differing probabilities of contracting the disease after first exposure or policies that treat exposed and susceptible individuals differently.

probability of infection is higher for an older individual than younger given the same exposure (14), consistent with decline in immune function with age. We adjust for this by letting the probability of infection be βp_{inf} when the exposed individual is over the age of 60, for $\beta > 1$. β is calibrated to match the fraction of deaths in China attributed to individuals over the age of 60, resulting in a value of 1.25. This is consistent with the relationship between age and attack rate amongst close contacts of a confirmed case reported by (14), where the increase in risk of infection for a contact over 65 years old was estimated in the range 1.12–1.92.

Sampling agents. Our process for sampling agents follows three steps that successively sample households, individual agents within households, and comorbidities for each agent. Because the full joint distributions over all of these quantities are not known, we implement a sampling procedure that respects the marginal distributions of household structure and age, as well as the marginal distribution for the occurrence of comorbidities within each age group.

First, we use information on the distribution of household structures to draw a type of household (e.g., single person, couple, nuclear family, or multigenerational family). Second, we sample the ages of the individual agents according to their role in the household (e.g., parent, child, or grandparent) combined with information about the age distribution of the population and the intergenerational interval. For China, we use household distributions from the 2010 Chinese census (15), intergenerational intervals from (16), and the age distribution provided by UN population statistics (17). For Italy, we use demographic statistics from Statista online portal about the following: household structure distribution (18), single-person households (19), couples with children (20) and corresponding family size (21), and single parents with children (22). Furthermore, we assume that children could stay within the family until the age of 30 and that couples without children were aged 30+, to account for societal patterns reported in familial studies which may have affected household distribution metrics (23). In New York City, we circumvent these steps by instead sampling individual households directly from census microdata. We use the public use microdata from the 2015 American Community Survey (24). We draw from household-level responses located in New York City, repeatedly sampling a household of individuals with their reported ages until the desired population size (8.4 million) is reached.

Third, we sample comorbidities from the corresponding country- and age-specific distributions. For China, we use estimates on age-specific prevalence of diabetes (25) and hypertension (26). For Italy, we use estimates from the Global Burden of Disease study on diabetes (3) and a recent study of age-stratified hypertension prevalence (27). For New York City, we use city-level estimates of age-specific prevalence for both comorbidities (28, 29). We ensure that diabetes and hypertension are appropriately correlated using a single global estimate for the probability of hypertension in individuals with diabetes (30). An important limitation of this study is that using different data sources for comorbidity prevalence in each location (while necessary) may introduce bias; our analyses could be refined if more comprehensive data sources became available.

Estimating disease progression from age and comorbidities. Many of the parameters for this model are assigned values based on estimates in the literature, shown in Table 1. However, we currently lack a detailed understanding of the joint impact of age and comorbidities on disease progression and mortality. Currently, estimated infection fatality rates (IFRs) are available by age but not for each specific combination of age and comorbidities. To obtain these specific estimates, we model the IFR with a logistic regression fit to IFRs estimated by Verity et al. (31) on data from mainland China. The logistic model is discussed in the next section. This model yields $p_{m\to d}(a_i, c_i)$, the country-independent probability that an individual i of age a_i and comborbidity status c_i will die if infected with SARS-CoV-2. Corrections for country-specific differences in mortality are handled via the parameter d_{mult}.

The simulation also requires specific values for the probabilities of transitioning between the disease states mild, severe, critical, and death. However, there is currently insufficient information available to infer the probabilities of these individual transitions for each combination of age and comorbidity. We assume that while the absolute values of these probabilities may vary based on age and comorbidity, the *ratios* between them do not exhibit such strong dependency. In particular, we assume that there are coefficients $\gamma_{s\to c}(a_i)$ and $\gamma_{c\to d}$ such that $p_{s\to c}(a_i, c_i) = \gamma_{s\to c}(a_i) p_{m\to s}(a_i, c_i)$ and $p_{c\to d}(a_i) = \gamma_{c\to d} p_{m\to s}(a_i, c_i)$. We allow $\gamma_{s\to c}(a_i)$ to be age-specific while assuming that $\gamma_{c\to d}$ is age-homogeneous because of the information currently available to estimate them; namely, we set $\gamma_{s\to c}(a_i)$ based on the estimated probabilities of hospitalization from (31) and ICU admission by age group in the US from (32) and $\gamma_{c\to d}$ based on the probability of death for all critical patients in China (33). Note that we assume both coefficients to be independent of the comorbodities c_i. Then, we can solve for $p_{m\to s}(a_i, c_i)$ such that

$$p_{m\to s}(a_i, c_i) \cdot \gamma_{s\to c}(a_i) p_{m\to s}(a_i, c_i) \cdot \gamma_{c\to d} p_{m\to s}(a_i, c_i) = p_{m\to d}(a_i, c_i),$$

and set $p_{s\to c}(a_i, c_i)$ and $p_{c\to d}(a_i, c_i)$ accordingly. Future work can relax the assumptions in this process as more information becomes available about how age and comorbidity impact the progression between disease states.

Estimating mortality from age and comorbidities. We require a model of $p_{m\to d}(a_i, c_i)$, however existing data sources only specify $p_{m\to d}(a_i)$ and $p_{m\to d}(c_i)$. To infer the joint distribution, we assume a linear (logistic) interaction between age bracket, diabetes status, and hypertension status. Specifically, we assume

$$p_{m\to d}(a_i, c_i) = \sigma\Big(\beta_{\mathrm{age}}(a_i) + \beta_{\mathrm{diabetes}} \mathbb{1}\left[\text{diabetes} \in c_i\right] + \beta_{\mathrm{hypertension}} \mathbb{1}\left[\text{hypertension} \in c_i\right]\Big),$$

where $\beta_{\mathrm{age}}(a_i)$ has a value for each age bracket (e.g., 20-30, 30-40, etc., 7 in total) and $\beta_{\mathrm{diabetes}}$ and $\beta_{\mathrm{hypertension}}$ are scalars.

The marginal distribution $p_{m\to d}(a_i)$ is taken from (31), which corrects for underreporting of infections in China. To obtain a comparable marginal distribution $p_{m\to d}(c_i)$, we scaled the reported CFR for each comorbidity group (33) by an age-adjusted correction for reporting obtained based on (31) (making the assumption that the probability of documentation is independent of comorbidity status after conditioning on age). We obtained data from the literature on the prevalence of diabetes and hypertension (26) in China by age (25), as well as a single global estimate of $p(\text{hypertension}|\text{diabetes})$ (30). We assume that these distributions are the same in COVID-19 patients as in the general population. However, we also conducted a sensitivity analysis to acknowledge the potential for increased comorbidity prevalence in COVID-19 patients, a scenario where comorbidities are also correlated to risk factors for transmission. The results (shown in Fig. S4) do not significantly alter our estimates. Given this information, we use gradient descent to find a set of parameters β which minimize the mean squared error in the following marginal consistency constraints:

$$p_{m\to d}(a_i) = \sum_{\text{diabetes},\text{hypertension}} p(\text{diabetes}, \text{hypertension}|a_i) p_{m\to d}(a_i, \text{diabetes}, \text{hypertension}), \quad \forall a_i,$$

$$p_{m\to d}(\text{diabetes}) = \sum_{a_i} p(a_i|\text{diabetes}) \sum_{\text{hypertension}} p(\text{hypertension}|a_i, \text{diabetes}) p_{m\to d}(a_i, \text{diabetes}, \text{hypertension}),$$

$$p_{m\to d}(\text{hypertension}) = \sum_{a_i} p(a_i|\text{hypertension}) \sum_{\text{diabetes}} p(\text{diabetes}|a_i, \text{hypertension}) p_{m\to d}(a_i, \text{diabetes}, \text{hypertension})$$

The set of estimated parameters are

$$\begin{aligned}
&\beta_{\text{age}}(18-30) = -8.49,\\
&\beta_{\text{age}}(30-40) = -7.68,\\
&\beta_{\text{age}}(40-50) = -7.41,\\
&\beta_{\text{age}}(50-60) = -6.39,\\
&\beta_{\text{age}}(60-70) = -5.41,\\
&\beta_{\text{age}}(70-80) = -4.54,\\
&\beta_{\text{age}}(80-100) = -4.05,\\
&\beta_{\text{diabetes}} = 1.22,\\
&\beta_{\text{hypertension}} = 1.58.
\end{aligned}$$

The coefficients should be interpreted relative to the baseline -8.49 value for the 18-30 group. For example, the value -7.68 for the 30-40 group indicates that the log-probability of mortality increases by 0.80 when age is increased from 18-30 to 30-40, holding comordibity status equal. Over 10 random restarts, the marginal values were always fit to within numerical tolerance by the same set of parameters (less than 0.1% maximum difference in the value of a parameter between runs). This suggests that the model parameters are fully identifiable in this setting.

Experimental settings

Experimental settings for Hubei. We draw a population of individuals from the age, household, and comorbidity distributions for China since more specific information is not available for Hubei (though the fraction of individuals over 65 is within the typical range for many Chinese provinces (34)). We simulate a population of 58.5 million individuals, matching the population of the Hubei province. After the lockdown, all contact frequencies are reduced by $\delta_c = 0.993$, set to obtain the number of outside-of-household contacts reported in post-lockdown surveys (14). Note that (14) reported a decline in 86.3% for total contacts, but this figure included within-household contact (which accounted for 94.1% of post-lockdown contact). We also modeled closure of schools on the lockdown date.

We set the range of the uniform prior distributions as follows. The prior over p_{inf} was set to contain all values with significant likelihood, with the final range being $[0.020, 0.035]$. The prior over t_0 was set to contain up to 7 days before the first reported case on November 17 (35), and 3 days afterwards (for a set of 10 days total). It is possible that substantial new backdating of the start of the epidemic could alter our results. Finally, the parameter d_{mult} captures variation in IFR, which is not precisely known in any location. We start from age-stratified IFR estimates by Verity et al. (31). In our model, these values result in an overall IFR of approximately 0.4% (lower than the 0.66% estimated by (31) because attack rates in our model are higher in younger groups, due to the larger numbers of daily contacts in younger groups vs older (9)). We placed a uniform prior over d_{mult} in the range [1,3], and then conditioned in the posterior on the IFR lying in the range 0.4–0.8%. Together, this procedure is designed to allow variation by approximately 50% around the IFR estimated by (31).

Experimental settings for Lombardy. We simulate a population of 10 million individuals (representing the population of Lombardy) drawn from the Italian distribution of age, household structure, and comorbidity status. The full demographic information needed to parameterize the simulation was not available for Lombardy specifically, but available information

suggests broadly similar characteristics (e.g., the median age in Lombardy is 45 (36), comparable to Italy in general at 46.5 (37)). After the lockdown on March 8, the number of contacts for all age groups is reduced to δ_c times its previous value. The prior for δ_c was uniform over the interval $[0, 0.1]$, reflecting a 90-100% reduction in outside of household contact (this interval was set to contain all values with significant likelihood). We also model closure of all schools on the lockdown date.

As in Hubei, we set the prior range for p_{inf} to include all values with significant likelihood, resulting in an interval [0.025, 0.04]. Also as in Hubei, the prior for t_0 was set to be uniform over a range of dates including up to 7 days before the infected travelers reportedly landed in Milan on January 23 (38), and up to 3 days afterwards. We adjusted the way the parameter d_{mult} was applied to account for the substantially different age composition of deaths in Italy than in either Hubei or New York City. Specifically, approximately 95% of reported deaths in Italy were among individuals 60 years or older, compared to approximately 80% in China (33) or 73.6% in New York City (39). One potential factor which could contribute to disparities in death rates are reports that older individuals in severe condition may have been less likely to receive care under the triage strategies adopted in response to overburdened health systems in Italy (40, 41). Accordingly, instead of scaling fatality rates uniformly across age groups, we calibrated a multiplier for the fatality rate in the 60+ age group to match the fraction of deaths attributed to that group.

Experimental settings for New York City. We simulate a population of 8.4 million individuals (representing the population of New York City), sampled in household units from census microdata for New York City.We model a two-step reduction in contact, consistent with mobility data (42, 43). The official lockdown was instituted on March 23, and we model a reduction in contact by δ_c on that date, with δ_c sampled uniformly from the interval [0, 0.1]. However, mobility data shows that significant reductions in mobility began the week before the official lockdown, suggesting preemptive distancing measures by individuals in anticipation of an official policy. Accordingly, we model contact in all non-household settings as reduced to 67% of its previous value starting on March 16. This factor was chosen based on the reduction in close physical encounters between cell phones represented in Unacast location data during the week of March 16 (43); we opted to fix this value instead of estimating a separate parameter in order to avoid increasing the number of free parameters in the model. Our estimated values for δ_c suggest that encounter rate data from mobile phones may be a reasonable proxy for the reduction in physical contacts; our posterior mean estimate for δ_c in New York City was 0.97, while Unacast encounter data showed a peak reduction in the encounter rate of 90-99% across the various boroughs of New York City.

We set the prior range for p_{inf} to be [0.03, 0.07], again set to include all values with non-negligible likelihood. d_{mult} was given a uniform prior over the range [1, 4], allowing for but not mandating a higher IFR than Hubei. We handled the starting conditions of the epidemic differently in New York City than in Lombardy due to reports of multiple distinct importation events over the course of February (44), with modeling studies suggesting the potential for thousands of cases present by the start of March (45). Instead of attempting to explicitly model multiple importations, we fixed t_0 at February 10 and placed a uniform prior over the number of infected individuals present on that date, in the range [5, 500].

Experimental settings for containment policies. We simulate two sets of scenarios modeling the impact of different containment policies. The first set of scenarios, shown in the main text, simulates a second-wave scenario for each location. We initialize the simulation to draws from the posterior distribution for said location. The posterior is over both the population-level model parameters, as well as the latent individual-level variables (whether each individual has been infected, etc.). Therefore, the fraction of individuals with (assumed) acquired immunity is also distributed according to the posterior. When the second-wave scenario starts for Lombardy and New York City, there is a low but non-zero level of circulation of the virus (approximately 100 actively infectious individuals). We initialize Hubei to a similar state, with 100 individuals newly infected at random when the scenario starts. In all locations, the modeled second-wave contact reductions are imposed at the start of the scenario when the number of infected individuals is low; this allows us to demonstrate the impact of the contact reductions as distinct from the cost of waiting to impose them. In order to simulate physical distancing by the entire population, we reduce the expected number of contacts M_{ij}^s between each pair of age groups i and j in setting s to $\delta_{\text{second}} M_{ij}^s$. Contacts within the household are unchanged. Our experiments examine $\delta_{\text{second}} \in [0.05, 0.25, 0.5, 0.75, 1]$. Physical distancing is also complemented by salutary sheltering by a single age group. For each age group, we simulate the impact of 25%, 50%, or 75% of the members of that age group sheltering (in addition to physical distancing at each level δ_{second} by the rest of the population). We run the simulation until the end of 2021 to ensure that the epidemic has had time to run its course in all scenarios and report the final median number of new infections and deaths for each scenario.

We also simulate a corresponding set of scenarios where the population begins in a completely susceptible state. For these simulations, population-level parameters are sampled from the posterior distribution as in the second-wave scenarios, but the population is initialized to be completely susceptible (apart from the randomly-sampled initially infectious individuals, as in the start of our experiments analyzing the first wave for each location). Contact reductions are imposed immediately, again to disentangle the impact of the reductions themselves on the number of expected infections from the cost of waiting to impose the intervention. We simulate the same set of combinations of physical distancing and salutary sheltering as above.

Mechanisms for physical distancing

Our analysis in Hubei, Lombardy, and New York City suggests that combining salutary sheltering by a portion of the population with physical distancing by the rest can be effective at mitigating the epidemic. In simulation, we modeled physical distancing as reducing the expected number of daily out-of-household contacts between any two members of the population (who are

not sheltering) by a factor of two. Here, we consider specific ways that this kind of physical distancing could be achieved by governments, businesses, and community organizations. We consider categories of daily activities defined by the American Time Use Survey (46) and draw on the literature around pandemic preparedness to provide concrete ways that the number of daily contacts can be reduced within each category. We note that this survey draws primarily on American sources. While many suggestions will be broadly applicable, similar sets of recommendations could be compiled specific to particular locations.

Work Businesses can take many actions to reduce the number of workers who are present at the workplace at a given time and to increase the amount of physical distance between workers who are present. Beyond the extreme solution of extended leaves of absence which could hurt the economy in the long run, more viable examples include have workers telecommute (even partially if their job cannot be performed fully remotely), staggering shifts to reduce the number of workers simultaneously present, increasing physical spacing between workers in an office, limiting the use of shared workstations, offering alternate locations to eat meals instead of a shared lunchroom, and staggering breaks and lunch hours (47, 48). "Cohorting" – keeping smaller groups of workers together over time so that contact remains mostly within the cohort – may also be helpful at reducing the total number of other people a given individual comes into contact with (47). (49) surveyed three epidemiological and twelve influenza-related modeling studies, showing that workplace physical distancing measures reduced the cumulative attack rate in the general population. They also established that epidemic peaks were delayed and reduced and that these positive effects were more pronounced when workplace physical distancing measures were coupled with other types of non-pharmaceutical interventions, as well as with therapeutic solutions. Means of instituting shift work, including staggering hours and compressed work-weeks, are reviewed by Totterdell (50), along with evidence about the health and wellness impacts of shift schedules on workers. Finally, an important consideration is availability of sick or personal leave so that symptomatic workers are able to isolate instead of continuing to expose coworkers (51). As (52) point out, and similar to school closure policies, both the timing and duration of workplace interventions would be critical to affect the course of the pandemic.

Commuting Adoption of shift work by businesses also has the potential to reduce physical contact on public transit. Apart from the scheduling of individual workers, staggering of business hours across a city – such that different businesses open and close at different times – was a tool used by the City of New York during the 1918 influenza pandemic to reduce congestion on public transit (53). Congestion pricing is another potential tool to reduce peak occupancy on public transit. It is worth noting that congestion pricing may entail equity concerns, which could be mitigated by charging different rates depending on income (54) or by implementing the program instead as a subsidy for using transit during off-peak hours.

Grocery shopping Several measures have been adopted by grocery stores during the COVID-19 pandemic to reduce the number of shoppers who come into contact with each other. These include making aisles one-way (55), limiting the number of individuals in the store at a given time (55), marking appropriate spacing on the floor in checkout lines (56), and reserving specific hours for members of a particular age group (e.g., older groups) (57). CDC pandemic preparedness guidelines also recommend that individuals reduce their frequency of trips to the grocery store (58). This could be instituted either on an individual/voluntary basis or via governments or stores assigning specific time periods for individuals or groups of individuals to shop. Some communities have also used volunteers to deliver groceries to individuals who are older or otherwise more vulnerable (59, 60).

Sports, exercise, and recreation Gyms could stagger the times that individuals use the facility and take steps to limit close contacts, e.g., ensuring appropriate spacing between people in a group exercise class. Improving ventilation in gyms may also be important to avoid increased transmission rates due to physical exertion in an enclosed space.

Attending or hosting social events Restaurants and bars could be required to maintain appropriate spacing between parties. Events involving more than a specific number of people could still be restricted.

Religious and spiritual activities In order to reduce the density of contacts at religious services, possible measures include increasing the number of services to reduce the number of people attending each service, cancelling associated social gatherings like coffee hours or youth groups, sending religious school lessons home with students instead of holding sessions in-person, broadcasting services where possible, and providing counseling services by phone instead of in person (48).

Feasibility of physical distancing. The feasibility of instituting proposed mechanisms for physical distancing may be subject to a range of considerations, including economic circumstances. This is particularly relevant for workplace physical distancing measures. We survey available information on several considerations related to the economic feasibility of physical distancing in different populations: telecommuting patterns, availability of sick leave, and consequences of reduced operations by a workplace.

Telecommuting. We consider the most recent statistics as provided by the 2017 State of Telecommuting Report about the U.S. employee workforce (61) to better understand the demographics of home-based workers. Some subsets of the adult population – depending on the age category, occupation type, and industry – were already more likely to work from home than others before this pandemics. Taking these factors into consideration helps rate the feasibility of our proposed policies and provides guidance for immediate intervention.

Age Half of telecommuters in the US are 45 years of age or older, as compared to just 41% of the overall workforce (61). Specifically, the greatest disproportional participation in home-based work is observed for the following age groups: 65+ years old (odds ratio, OR=1.7) and 55-64 years old (OR=1.2).

Occupation type Among telecommuting jobs, sales, management, office, and administrative occupations are the most common. Together, they account for 43% of home-based jobs, while representing only 34% of non-telecommuting occupations. Five type of occupations are more prevalent among home-based workers as opposed to non-telecommuting workforce participants (61): computer and mathematical work (OR=2.8), military work (OR=2.5), arts/design/entertainment/sports/media (OR=2.0), personal care and service (OR=2.0), and business and financial operations (OR=1.9).

Industry The largest share of telecommuters is due to three industries (61): professional, scientific, and technical services (17%); healthcare and social assistance (11.6%); and finance and insurance (9.7%). The prevalence in telecommuting vs. non-telecommuting work is higher in management (OR=32.8), agriculture (OR=3.4), real estate (OR=3), information (OR=3), and mining (OR=2.4).

Access to telecommuting options In the US, having the possibility to telecommute is mostly a function of both the worker's company size and employment status. Telecommuting is more prevalent among large vs. small companies. 12% of firms with more than 500 employees offer such a possibility, whereas only 5% of those with less than 100 employees do so. Moreover, employers are more likely to offer the option to work from home to their full-time rather than part-time employees (8% vs. 2% respectively as of 2017). It is expected that these tendencies would be confirmed in other industrialized countries as well.

Availability of sick leave. Policies related to sick leave vary widely by location. For example, in the United States, workers are not generally guaranteed paid sick leave; only 11 states require employers to offer workers paid leave (62). As a consequence, 24% have no paid sick leave at all, with strong heterogeneities in access to paid sick leave by income level, occupation type, and activity sector (63). Notably, 93% of workers in the top tenth of the income distribution receive paid sick leave, compared with only 30% of those in the bottom tenth (62). However, the adoption of isolation of potentially infected individuals is strongly contingent on access to paid sick or personal leave.

Consequences of reduced operations. For work that cannot be performed remotely, reduced operations due to sheltering or physical distancing interventions will inevitably reduce employment. For a less than total shutdown, work in the labor economics literature considers strategies to allocate the reduced number of hours available to workers that have not been affected by lay-offs, including the use of a minimum hours threshold corresponding with guaranteed earnings (64), as well as a maximum number of hours worked per person to preclude additional employment inequalities. Regardless, additional options to offset the income loss for eligible workers include provisions for unemployment insurance (65).

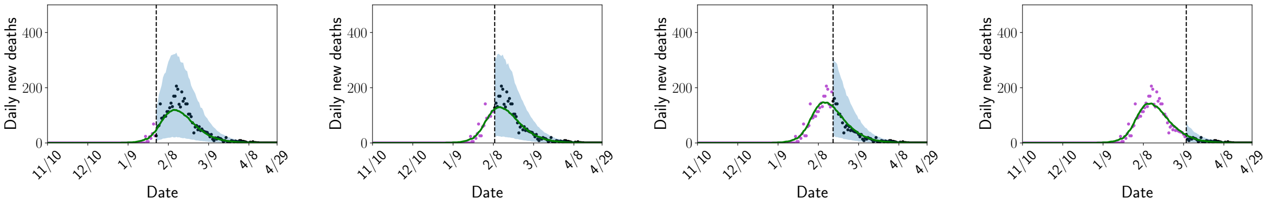

Fig. S1. Predictive posterior for Hubei as a function of when the training period ends. Black dashed line: end of training period. Green line: posterior median. Blue shaded region: 90% credible interval. Pink dots: training data. Black dots: held-out data. The 90% credible interval of the predictive posterior contains the held-out data at all points, including when the model is fit using only data from the earliest portion of the epidemic.

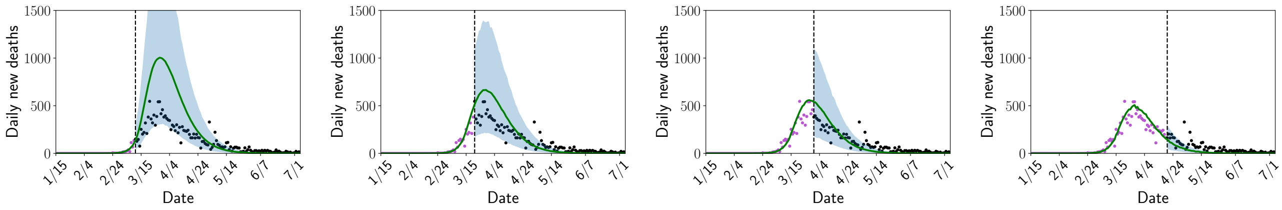

Fig. S2. Predictive posterior for Lombardy as a function of when the training period ends. Black dashed line: end of training period. Green line: posterior median. Blue shaded region: 90% credible interval. Pink dots: training data. Black dots: held-out data. The 90% credible interval of the predictive posterior includes contains the held-out data at almost all points, including when the model is fit using only data from the earliest portion of the epidemic. The model over-predicts deaths early in the epidemic, though the timing of the peak is correctly captured early on. Much of the over-prediction is corrected with additional training data even before the peak is observed.

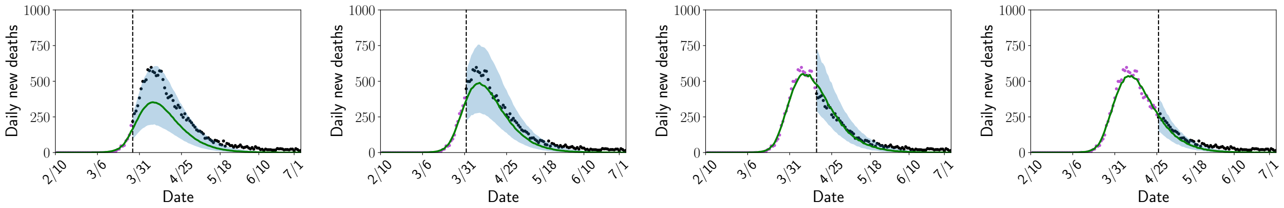

Fig. S3. Predictive posterior for New York City as a function of when the training period ends. Black dashed line: end of training period. Green line: posterior median. Blue shaded region: 90% credible interval. Pink dots: training data. Black dots: held-out data. The 90% credible interval of the predictive posterior includes contains the held-out data at all points, including when the model is fit using only data from the earliest portion of the epidemic. Using data from only the earliest stage, the model slightly misidentifies the timing and magnitude of the peak, but these aspects of the prediction substantially improve even without observing the peak in the training data (c.f. the first vs second figure from the left).

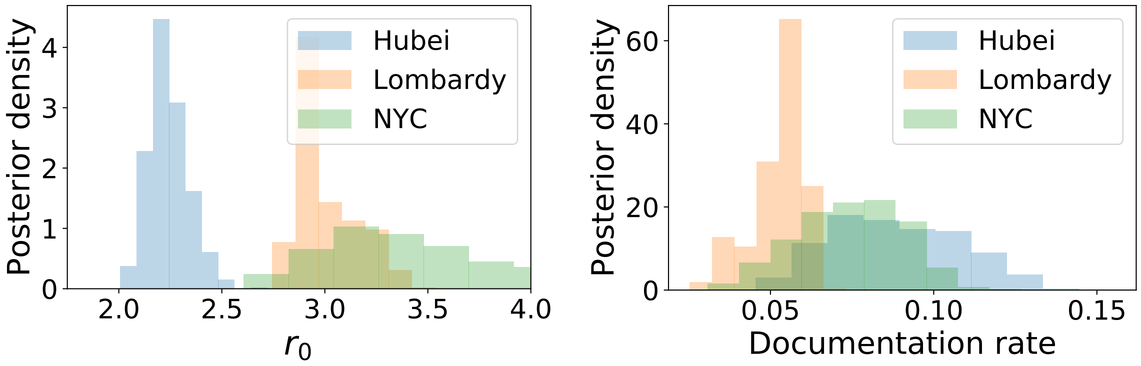

Fig. S4. Sensitivity analysis to higher prevalence of comorbidities in the population of COVID-19 patients use to infer the age and comorbidity-specific infection fatality rate. Each plot shows the posterior distribution over a given quantity (left: the basic reproductive number r_0; right: the documentation rate for infections) for each location. In this scenario, the regression coefficients $\beta_{\text{age}}, \beta_{\text{diabetes}}, \beta_{\text{hypertension}}$ which produce $p_{m \to d}(a_i, c_i)$ (the probability of death given age a_i and comorbidities c_i) are estimated assuming that the prevalence of both diabetes and hypertension are twice as high in the COVID-19 patients in China for whom case fatality rates are available as in the general population for China. Our main analysis assumed equal prevalence in COVID-19 patients as in the general population. Our major conclusions are unaltered.

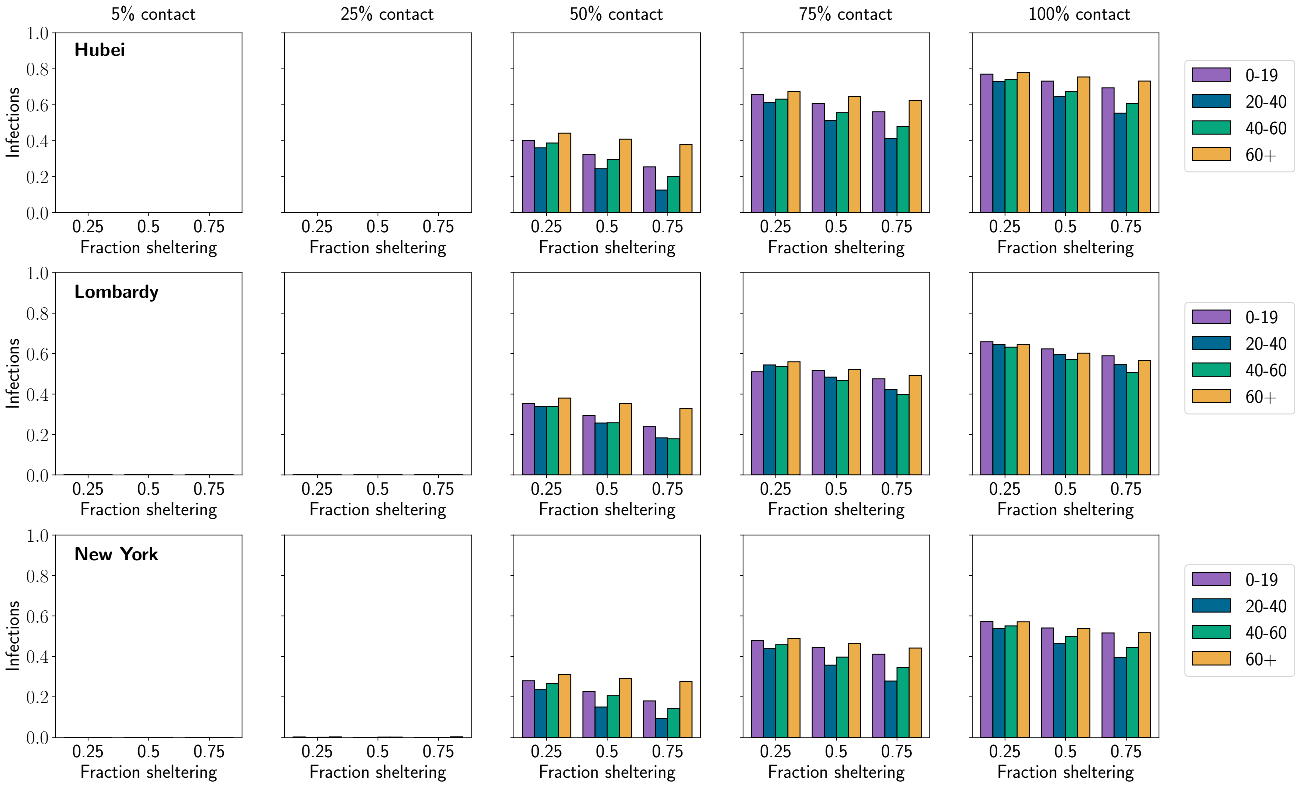

Fig. S5. Fraction of the population newly infected in each location in each hypothetical second-wave scenario. Each row shows the results for the specified location, while each column shows a given level of physical distancing by the entire population (specified as the percentage of normal contact levels). The x-axis within each figure gives the fraction of a single age group which adopts salutary sheltering. Each bar represents a scenario where the given fraction of a single age group adopts salutary sheltering, with the color of the bar representing the identity of the group (see legend). We find that for all populations, 25% or less contact is sufficient to suppress the epidemic. At 50% contact, a significant portion of each population becomes infected (approximately 10-40% depending on the population, which group shelters, and what fraction of that group shelters). Across populations, sheltering by the 20-40 and 40-60 age groups reduces infections by the largest amount; sheltering by the 60+ group has only a minor impact.

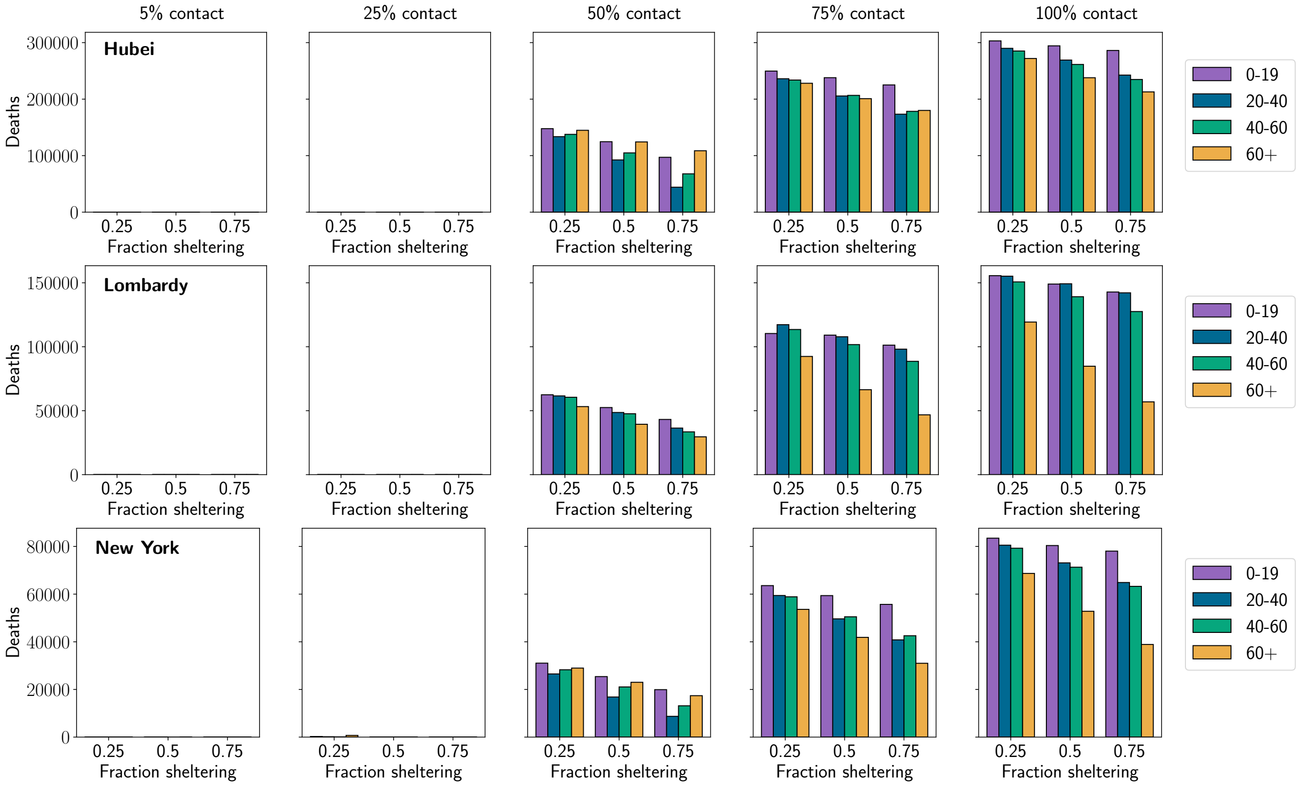

Fig. S6. Number of deaths in each population in each hypothetical second-wave scenario. Each row shows the results for the specified location, while each column shows a given level of physical distancing by the entire population (specified as the percentage of normal contact levels). The x-axis within each figure gives the fraction of a single age group which adopts salutary sheltering. Each bar represents a scenario where the given fraction of a single age group adopts salutary sheltering, with the color of the bar representing the identity of the group (see legend). In scenarios with 25% or less contact, the outbreak is effectively surpressed (see Figure S5) resulting in correspondingly few deaths. At 50% contact, the larger number of infections results in a larger number of deaths. For Hubei and New York City at 50% contact, deaths are reduced more effectively via sheltering by the 20-40 or 40-60 groups than by the 60+ group. In Lombardy, sheltering by the 60+ group is always the most effective at reducing deaths but the margin between the number of deaths under sheltering by the 60+ group compared to other groups is smaller under 50% contact than under higher contact levels. At 75% or higher contact, this pattern is replicated in Hubei and New York City, where sheltering by the 60+ group has the greatest marginal impact on deaths and the gap between the 60+ and other groups is larger at 100% contact than at 75%.

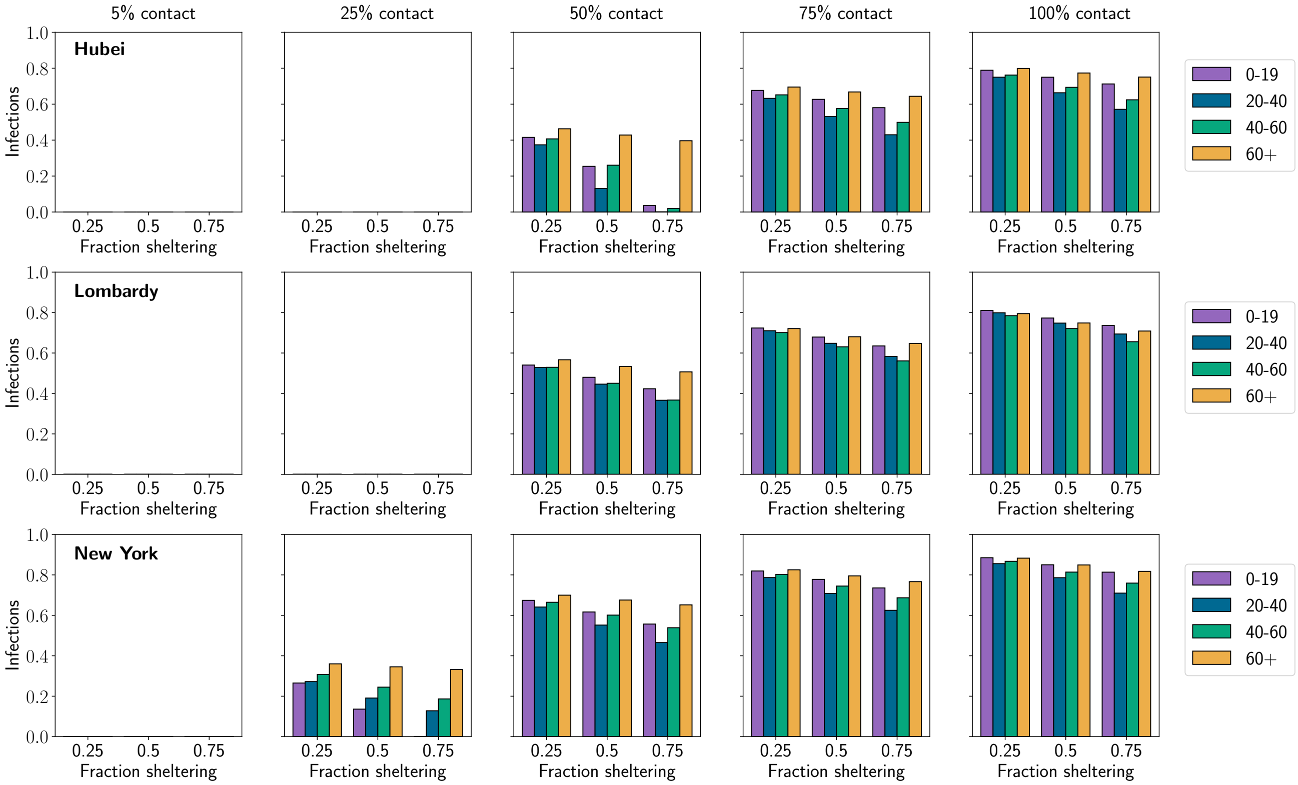

Fig. S7. Fraction of the population infected in each population in each hypothetical scenario with a completely susceptible population. Each row shows the results for the specified location, while each column shows a given level of physical distancing by the entire population (specified as the percentage of normal contact levels). The x-axis within each figure gives the fraction of a single age group which adopts salutary sheltering. Each bar represents a scenario where the given fraction of a single age group adopts salutary sheltering, with the color of the bar representing the identity of the group (see legend). In contrast to the second-wave scenarios shown in Figure S5, 25% contact is not always sufficient to suppress a widespread outbreak. This reflects two factors. First, the importance of acquired immunity accumulated during the first outbreak in reducing the effective reproduction number. Second, the potential for the timing of when interventions are applied to influence the total extent of infections. E.g., it is possible for the total number of eventual infections to be lower when more people are infected in the first wave than when more stringent control measures are imposed from the start (66). At 50% contact and above, the dynamics become more similar to the second-wave scenarios, with substantial fractions of each population infected. As in Figure S5, sheltering by the 20-40 and 40-60 age groups reduces infections by the largest amount; sheltering by the 60+ group has only a minor impact.

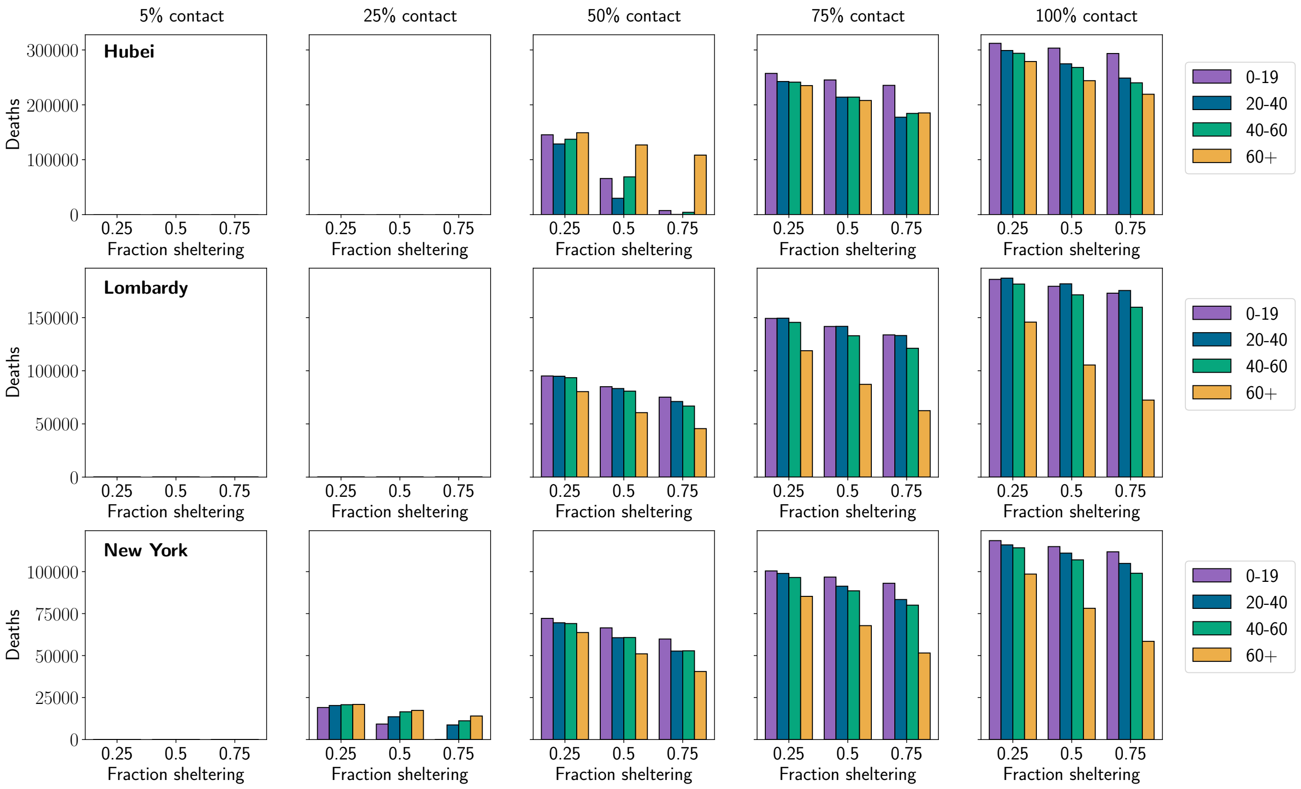

Fig. S8. Number of deaths in each population in each hypothetical scenario with a completely susceptible population. Each row shows the results for the specified location, while each column shows a given level of physical distancing by the entire population (specified as the percentage of normal contact levels). The x-axis within each figure gives the fraction of a single age group which adopts salutary sheltering. Each bar represents a scenario where the given fraction of a single age group adopts salutary sheltering, with the color of the bar representing the identity of the group (see legend). Deaths are limited by contact levels at 25% or lower. As in the second-wave scenarios for Hubei and New York City, at low levels of contact, sheltering by the 20-40 and 40-60 age groups is more effective at reducing deaths than sheltering by the 60+ group. However, due to the larger number of infections at a given contact level in the completely-susceptible scenario as compared to the second-wave scenario, a lower level of overall contact is sometimes needed to realize this effect (25% contact in New York City instead of 50%). Once contact levels rise to 50%, only Hubei shows greater effectiveness for sheltering by the 20-40 and 40-60 age groups, and at 75% contact it is more effective for the 60+ age group to shelter for all populations.

Table S1. Model parameters

Parameter	Description	Value and/or source
$p_{m\to s}(a_i, c_i)$	Prob. of progressing from mild to severe given age a_i and comorbidities c_i	Estimated from (31) (see above)
$p_{s\to c}(a_i, c_i)$	Prob. of progressing from severe to critical given age a_i and comorbidities c_i	As above
$p_{c\to d}(a_i, c_i)$	Prob. of progressing from critical to death given age a_i and comorbidities c_i	As above
p_h	Prob. of infecting each household member each day	Calibrated to match (13)
p_{inf}	Prob. of infecting an outside household contact	Free parameter
$\mu_{e\to m}$	Log-mean time to progress from exposed to mild (mean incubation period)	1.621 (12)
$\sigma^2_{e\to m}$	Log-standard deviation time to progress from exposed to mild	0.418 (12)
$\lambda_{m\to s}$	Mean time to progress from mild to severe	7 days (67)
$\lambda_{s\to c}$	Mean time to progress from severe to critical	7.5 days (using 14.5 days from onset to mechanical ventilation in (4))
$\lambda_{c\to d}$	Mean time to progress from critical to death	4.5 days (subtracting $\lambda_{m\to s}$ and $\lambda_{s\to c}$ from onset-to-death in (4))
λ_{isolate}	Mean time for an individual in the mild state to isolate	4.6 days (time to first medical care (68))
$\lambda_{m\to r}$	Mean time to recovery for an individual in the mild state	14 days (67)
$\lambda_{s\to r}$	Mean time to recovery for an individual in the severe state	$28 - \lambda_{m\to s}$ (midpoint of onset-to-recovery for severe (67))
$\lambda_{c\to r}$	Mean time to recovery for an individual in the critical state	$35 - \lambda_{m\to s} - \lambda_{s\to c}$ (midpoint of (67) onset-to-recovery for critical)
α	Reduction in infectiousness before symptoms	0.55 (8) †
M	Contact matrix (for each country)	(9)
t_0	First date with at least 5 infected individuals	Free parameter

† This setting for α is likely pessimistic in that Li et al.'s estimate for reduction in transmissibility is for undocumented cases, including asymptomatic cases, presymptomatic cases, and those with limited symptoms (8). Future work should examine the impact of a potentially lower α as better information on transmissibility in the asymptomatic or presymptomatic state becomes available.

Table S2. Comparison of Poisson and negative binomial observation models in each location, along with estimated dispersion parameter σ^2_{obs} for the negative binomial. The negative binomial model is strongly preferred by AIC in each location. S2

Location	Poisson AIC	Negative binomial AIC	σ^2_{obs}
Hubei	891.30	670.26	0.337
Lombardy	4741.82	877.97	0.278
New York City	657.49	533.26	0.0641

Table S3. Infections (in thousands) for a second-wave scenario in Hubei. Each major row heading denotes the age group which adopts salutary sheltering, and the sub-headings denote the fraction of the group which shelters. The major column headings give the level of contact amongst individuals who do not shelter. The entry "Total" gives the median number of total infections (in thousands) in each scenario, while "0-59" and "60+" give the median number of total infections in each segment of the population (under or over 60 years of age).

	5% contact			25% contact			50% contact			75% contact			100% contact		
	Total	0-59	60+	Total	0-59	60+	Total	0-59	60+	Total	0-59	60+	Total	0-59	60+
0-19															
25%	0.2	0.2	0.0	1.0	1.0	0.1	23445.4	20439.3	3006.0	38372.9	33291.2	5081.7	45077.8	38866.5	6211.4
50%	0.2	0.2	0.0	0.6	0.5	0.1	19004.6	16514.2	2490.4	35487.6	30666.7	4820.9	42811.7	36778.4	6033.2
75%	0.2	0.2	0.0	0.6	0.5	0.1	14888.0	12894.7	1993.4	32813.8	28264.0	4549.8	40609.1	34761.2	5847.8
20-40															
25%	0.2	0.2	0.0	0.6	0.5	0.1	21084.8	18314.8	2770.0	35816.7	30952.8	4864.0	42720.3	36677.2	6043.2
50%	0.2	0.2	0.0	0.5	0.4	0.1	14269.7	12289.0	1980.7	29955.1	25636.9	4318.2	37719.7	32089.5	5630.2
75%	0.2	0.2	0.0	0.5	0.4	0.1	7348.9	6332.3	1016.7	24057.7	20378.1	3679.6	32381.1	27224.4	5156.7
40-60															
25%	0.2	0.2	0.0	0.6	0.5	0.1	22662.4	19752.0	2910.3	36943.6	32022.0	4921.6	43409.4	37362.4	6046.9
50%	0.2	0.2	0.0	0.6	0.6	0.1	17298.6	15009.9	2288.7	32505.8	28035.0	4470.8	39484.8	33801.4	5683.5
75%	0.2	0.2	0.0	0.5	0.4	0.1	11798.9	10240.3	1558.6	28086.9	24099.4	3987.5	35458.4	30183.9	5274.5
60+															
25%	0.2	0.2	0.0	0.5	0.4	0.1	25872.8	22997.9	2874.9	39481.7	35000.5	4481.2	45658.2	40315.6	5342.6
50%	0.2	0.2	0.0	0.7	0.7	0.1	23916.2	21538.6	2377.6	37869.2	34094.9	3774.3	44153.0	39680.5	4472.4
75%	0.2	0.2	0.0	0.5	0.4	0.1	22211.0	20213.5	1997.6	36441.5	33227.7	3213.8	42824.5	39048.6	3776.0

Table S4. Infections (in thousands) for a second-wave scenario in Lombardy. Each major row heading denotes the age group which adopts salutary sheltering, and the sub-headings denote the fraction of the group which shelters. The major column headings give the level of contact amongst individuals who do not shelter. The entry "Total" gives the median number of total infections (in thousands) in each scenario, while "0-59" and "60+" give the median number of total infections in each segment of the population (under or over 60 years of age).

	5% contact			25% contact			50% contact			75% contact			100% contact		
	Total	0-59	60+	Total	0-59	60+	Total	0-59	60+	Total	0-59	60+	Total	0-59	60+
0-19															
25%	0.0	0.0	0.0	0.7	0.7	0.0	3540.9	2822.6	718.2	5101.1	3899.6	1201.4	6584.6	4945.4	1639.2
50%	0.0	0.0	0.0	0.3	0.3	0.0	2930.2	2319.9	610.3	5159.4	3955.0	1204.4	6234.3	4650.6	1583.6
75%	0.0	0.0	0.0	0.1	0.1	0.0	2409.4	1894.3	515.2	4753.2	3621.3	1132.0	5890.6	4363.8	1526.8
20-40															
25%	0.0	0.0	0.0	0.2	0.2	0.0	3376.0	2685.4	690.6	5440.0	4178.6	1261.4	6453.4	4823.1	1630.2
50%	0.0	0.0	0.0	0.2	0.2	0.0	2567.9	2014.8	553.2	4838.4	3671.2	1167.2	5962.5	4398.2	1564.3
75%	0.0	0.0	0.0	0.2	0.1	0.0	1832.9	1417.9	415.0	4218.4	3157.0	1061.4	5460.6	3963.9	1496.6
40-60															
25%	0.1	0.1	0.0	0.5	0.5	0.0	3377.9	2688.8	689.1	5353.9	4117.8	1236.2	6320.6	4724.2	1596.3
50%	0.0	0.0	0.0	0.3	0.3	0.0	2578.5	2028.5	550.0	4683.3	3565.5	1117.8	5702.0	4210.4	1491.6
75%	0.0	0.0	0.0	0.1	0.1	0.0	1786.5	1388.5	398.0	3985.8	2997.1	988.7	5065.0	3691.0	1374.0
60+															
25%	0.1	0.1	0.0	0.3	0.3	0.0	3800.9	3192.0	608.9	5593.7	4580.7	1013.0	6448.3	5170.1	1278.2
50%	0.1	0.1	0.0	0.4	0.3	0.0	3524.4	3069.0	455.5	5219.7	4484.1	735.6	6022.9	5104.1	918.8
75%	0.0	0.0	0.0	0.3	0.3	0.0	3297.4	2965.2	332.2	4927.6	4406.4	521.2	5664.5	5036.6	627.8

Table S5. Infections (in thousands) for a second-wave scenario in New York City. Each major row heading denotes the age group which adopts salutary sheltering, and the sub-headings denote the fraction of the group which shelters. The major column headings give the level of contact amongst individuals who do not shelter. The entry "Total" gives the median number of total infections (in thousands) in each scenario, while "0-59" and "60+" give the median number of total infections in each segment of the population (under or over 60 years of age).

	5% contact			25% contact			50% contact			75% contact			100% contact		
	Total	0-59	60+	Total	0-59	60+	Total	0-59	60+	Total	0-59	60+	Total	0-59	60+
0-19															
25%	0.0	0.0	0.0	12.4	0.1	0.0	2345.5	2020.6	336.1	4028.3	3376.4	678.1	4805.6	3948.3	895.2
50%	0.0	0.0	0.0	0.1	0.1	0.0	1906.2	1587.8	283.6	3718.9	3103.3	639.2	4539.5	3735.0	861.1
75%	0.0	0.0	0.0	0.1	0.0	0.0	1509.2	1194.1	236.9	3451.1	2875.9	607.4	4333.1	3529.0	827.9
20-40															
25%	0.0	0.0	0.0	0.1	0.1	0.0	1995.9	1752.2	293.6	3692.0	3072.5	640.8	4511.5	3678.4	869.5
50%	0.0	0.0	0.0	0.2	0.0	0.0	1256.4	1101.8	192.7	2996.8	2477.3	550.3	3906.4	3153.7	796.9
75%	0.0	0.0	0.0	0.0	0.0	0.0	770.0	484.4	90.0	2335.8	1907.2	449.4	3309.8	2605.5	716.1
40-60															
25%	0.0	0.0	0.0	3.5	0.1	0.0	2242.3	1966.4	317.5	3841.8	3218.4	649.5	4625.7	3788.1	868.9
50%	0.0	0.0	0.0	8.1	0.1	0.0	1723.8	1510.8	242.8	3329.8	2821.9	573.5	4194.0	3418.0	800.5
75%	0.0	0.0	0.0	0.2	0.0	0.0	1190.7	1068.7	172.2	2890.6	2420.0	494.5	3732.4	3033.8	727.8
60+															
25%	0.0	0.0	0.0	15.0	0.2	0.0	2610.8	2350.1	296.3	4098.8	3587.1	547.6	4794.9	4118.3	709.2
50%	0.0	0.0	0.0	7.8	0.2	0.0	2449.3	2277.8	216.4	3885.0	3525.2	395.4	4525.1	4085.0	509.3
75%	0.0	0.0	0.0	17.5	0.2	0.0	2315.0	2222.5	146.3	3706.2	3489.7	261.3	4340.5	4054.0	328.7

Table S6. Infections (in thousands) for a fully susceptible population in Hubei. Each major row heading denotes the age group which adopts salutary sheltering, and the sub-headings denote the fraction of the group which shelters. The major column headings give the level of contact amongst individuals who do not shelter. The entry "Total" gives the median number of total infections (in thousands) in each scenario, while "0-59" and "60+" give the median number of total infections in each segment of the population (under or over 60 years of age).

	5% contact			25% contact			50% contact			75% contact			100% contact		
	Total	0-59	60+	Total	0-59	60+	Total	0-59	60+	Total	0-59	60+	Total	0-59	60+
0-19															
25%	0.0	0.0	0.0	0.0	0.0	0.0	24296.0	21144.8	3151.2	39589.1	34295.6	5290.0	46137.4	39735.7	6400.5
50%	0.0	0.0	0.0	0.0	0.0	0.0	14864.2	12914.7	1925.4	36673.5	31651.5	5021.6	43865.4	37640.4	6226.8
75%	0.0	0.0	0.0	0.0	0.0	0.0	2139.0	1864.0	275.0	33972.3	29221.6	4750.7	41671.1	35624.3	6045.5
20-40															
25%	0.0	0.0	0.0	0.0	0.0	0.0	21874.8	18960.6	2911.8	36995.8	31921.2	5074.6	43873.1	37635.5	6237.6
50%	0.0	0.0	0.0	0.0	0.0	0.0	7641.0	6596.3	1042.1	31112.7	26597.1	4515.6	38817.0	32984.3	5833.9
75%	0.0	0.0	0.0	0.0	0.0	0.0	76.2	65.3	10.9	25148.4	21264.5	3884.0	33425.8	28073.7	5352.1
40-60															
25%	0.0	0.0	0.0	0.0	0.0	0.0	23800.0	20699.0	3101.1	38133.0	33002.5	5130.0	44565.3	38323.4	6242.3
50%	0.0	0.0	0.0	0.0	0.0	0.0	15228.0	13203.5	2024.6	33698.1	29020.9	4677.9	40584.2	34705.1	5879.5
75%	0.0	0.0	0.0	0.0	0.0	0.0	1167.4	1012.5	154.8	29187.1	25007.1	4182.1	36522.3	31045.5	5474.0
60+															
25%	0.0	0.0	0.0	0.0	0.0	0.0	27094.9	24043.3	3055.5	40666.9	35999.9	4668.2	46742.0	41214.4	5525.9
50%	0.0	0.0	0.0	0.0	0.0	0.0	25051.3	22519.2	2532.0	39071.9	35121.5	3948.6	45234.7	40594.2	4640.5
75%	0.0	0.0	0.0	0.0	0.0	0.0	23206.5	21080.0	2125.5	37662.2	34283.1	3379.0	43916.8	39987.2	3928.6

Table S7. Infections (in thousands) for a fully susceptible population in Lombardy. Each major row heading denotes the age group which adopts salutary sheltering, and the sub-headings denote the fraction of the group which shelters. The major column headings give the level of contact amongst individuals who do not shelter. The entry "Total" gives the median number of total infections (in thousands) in each scenario, while "0-59" and "60+" give the median number of total infections in each segment of the population (under or over 60 years of age).

	5% contact			25% contact			50% contact			75% contact			100% contact		
	Total	0-59	60+	Total	0-59	60+	Total	0-59	60+	Total	0-59	60+	Total	0-59	60+
0-19															
25%	0.0	0.0	0.0	0.4	0.3	0.1	5404.4	4367.2	1037.1	7237.4	5651.9	1585.5	8101.9	6172.4	1929.4
50%	0.0	0.0	0.0	0.2	0.2	0.0	4797.6	3860.1	937.5	6789.2	5274.0	1515.2	7730.4	5854.6	1875.8
75%	0.0	0.0	0.0	0.2	0.1	0.0	4232.5	3394.8	837.7	6351.7	4909.7	1442.0	7362.5	5542.4	1820.1
20-40															
25%	0.0	0.0	0.0	0.7	0.6	0.1	5281.9	4257.8	1024.1	7102.3	5525.1	1577.2	7991.1	6061.2	1929.9
50%	0.0	0.0	0.0	0.3	0.2	0.1	4459.5	3569.8	889.7	6477.8	4988.6	1489.2	7478.7	5608.5	1870.2
75%	0.0	0.0	0.0	0.1	0.1	0.0	3661.7	2906.6	755.1	5830.4	4439.6	1390.8	6941.8	5135.0	1806.9
40-60															
25%	0.0	0.0	0.0	0.4	0.3	0.1	5291.7	4270.3	1021.3	7009.6	5460.7	1548.8	7846.8	5953.5	1893.3
50%	0.0	0.0	0.0	0.2	0.2	0.0	4502.3	3610.8	891.5	6307.4	4878.0	1429.5	7207.5	5413.4	1794.1
75%	0.0	0.0	0.0	0.2	0.2	0.0	3672.9	2927.1	745.8	5611.5	4303.2	1308.4	6558.6	4870.6	1688.0
60+															
25%	0.0	0.0	0.0	1.4	1.2	0.2	5666.3	4782.8	883.5	7206.9	5933.0	1273.9	7947.3	6413.2	1534.1
50%	0.0	0.0	0.0	1.2	1.0	0.1	5331.1	4657.6	673.5	6804.6	5851.6	953.1	7486.4	6352.8	1133.6
75%	0.0	0.0	0.0	1.1	1.0	0.1	5069.1	4559.4	509.7	6469.1	5778.6	690.5	7088.0	6293.4	794.6

Table S8. Infections (in thousands) for a fully susceptible population in New York City. Each major row heading denotes the age group which adopts salutary sheltering, and the sub-headings denote the fraction of the group which shelters. The major column headings give the level of contact amongst individuals who do not shelter. The entry "Total" gives the median number of total infections (in thousands) in each scenario, while "0-59" and "60+" give the median number of total infections in each segment of the population (under or over 60 years of age).

	5% contact			25% contact			50% contact			75% contact			100% contact		
	Total	0-59	60+	Total	0-59	60+	Total	0-59	60+	Total	0-59	60+	Total	0-59	60+
0-19															
25%	0.0	0.1	0.0	2227.5	1988.4	239.2	5661.5	4875.9	785.8	6883.3	5796.8	1086.7	7433.6	6172.3	1261.7
50%	0.0	0.1	0.0	1142.8	1016.2	126.7	5179.5	4450.8	728.9	6532.9	5487.2	1045.9	7140.4	5907.8	1233.0
75%	0.0	0.1	0.0	1.6	1.5	0.2	4677.4	4010.6	667.0	6178.6	5174.0	1004.8	6833.1	5633.0	1200.5
20-40															
25%	0.0	0.1	0.0	2287.6	2045.7	242.0	5385.0	4626.6	758.6	6608.1	5540.0	1068.3	7186.9	5936.3	1251.0
50%	0.0	0.1	0.0	1603.4	1434.4	169.1	4635.2	3958.7	676.6	5944.9	4942.4	1002.8	6603.3	5399.4	1204.2
75%	0.0	0.1	0.0	1075.4	962.8	112.7	3908.1	3323.9	584.3	5247.3	4321.6	925.9	5962.6	4815.2	1147.7
40-60															
25%	0.0	0.1	0.0	2583.7	2314.0	269.8	5581.2	4814.0	767.4	6738.8	5672.3	1066.8	7282.6	6036.5	1246.5
50%	0.0	0.1	0.0	2055.8	1845.9	210.0	5049.1	4353.7	695.6	6256.0	5251.8	1004.4	6836.2	5641.0	1195.5
75%	0.0	0.1	0.0	1566.9	1411.0	155.5	4525.0	3904.7	620.4	5769.3	4834.9	934.6	6378.8	5240.0	1139.2
60+															
25%	0.0	0.1	0.0	3024.7	2759.0	265.9	5876.7	5218.4	658.5	6932.3	6047.8	884.7	7414.8	6393.5	1021.9
50%	0.0	0.1	0.0	2901.6	2695.9	205.7	5675.6	5177.9	497.9	6679.6	6018.9	661.1	7133.6	6373.8	760.3
75%	0.0	0.1	0.0	2786.6	2632.8	153.9	5475.6	5124.3	351.4	6439.2	5987.3	452.2	6865.7	6353.9	512.3

Table S9. Deaths (in thousands) for a second-wave scenario in Hubei. Each major row heading denotes the age group which adopts salutary sheltering, and the sub-headings denote the fraction of the group which shelters. The major column headings give the level of contact amongst individuals who do not shelter. The entry "Total" gives the median number of total deaths (in thousands) in each scenario, while "0-59" and "60+" give the median number of total deaths in each segment of the population (under or over 60 years of age).

	5% contact			25% contact			50% contact			75% contact			100% contact		
	Total	0-59	60+	Total	0-59	60+	Total	0-59	60+	Total	0-59	60+	Total	0-59	60+
0-19															
25%	0.0	0.0	0.0	0.0	0.0	0.0	147.8	29.4	118.1	249.7	50.4	198.2	303.2	61.2	239.6
50%	0.0	0.0	0.0	0.0	0.0	0.0	124.7	24.7	97.7	238.0	48.6	186.7	294.4	60.0	232.2
75%	0.0	0.0	0.0	0.0	0.0	0.0	97.1	19.8	76.9	225.2	45.9	175.9	286.2	58.7	225.4
20-40															
25%	0.0	0.0	0.0	0.0	0.0	0.0	133.6	24.9	108.0	236.2	45.4	188.2	290.0	56.3	230.7
50%	0.0	0.0	0.0	0.0	0.0	0.0	92.5	16.0	75.3	205.7	37.7	164.8	269.2	50.5	216.6
75%	0.0	0.0	0.0	0.0	0.0	0.0	44.1	6.8	35.5	173.4	29.9	142.2	242.6	42.9	197.2
40-60															
25%	0.0	0.0	0.0	0.0	0.0	0.0	137.7	24.3	113.3	233.9	41.9	191.6	285.3	50.8	231.5
50%	0.0	0.0	0.0	0.0	0.0	0.0	104.9	16.3	87.6	206.7	32.4	170.5	261.5	40.9	217.6
75%	0.0	0.0	0.0	0.0	0.0	0.0	67.8	9.1	57.1	178.5	24.2	151.4	234.9	31.5	202.8
60+															
25%	0.0	0.0	0.0	0.0	0.0	0.0	144.9	30.8	113.3	228.2	50.2	176.3	272.0	60.8	209.9
50%	0.0	0.0	0.0	0.0	0.0	0.0	124.3	28.5	95.6	200.9	48.6	151.5	237.9	58.9	177.6
75%	0.0	0.0	0.0	0.0	0.0	0.0	108.7	26.3	81.7	180.1	46.6	131.7	212.9	57.3	154.4

Table S10. Deaths (in thousands) for a second-wave scenario in Lombardy. Each major row heading denotes the age group which adopts salutary sheltering, and the sub-headings denote the fraction of the group which shelters. The major column headings give the level of contact amongst individuals who do not shelter. The entry "Total" gives the median number of total deaths (in thousands) in each scenario, while "0-59" and "60+" give the median number of total deaths in each segment of the population (under or over 60 years of age).

	5% contact			25% contact			50% contact			75% contact			100% contact		
	Total	0-59	60+	Total	0-59	60+	Total	0-59	60+	Total	0-59	60+	Total	0-59	60+
0-19															
25%	0.0	0.0	0.0	0.0	0.0	0.0	62.4	3.1	62.6	110.4	4.6	107.8	155.6	5.9	152.2
50%	0.0	0.0	0.0	0.0	0.0	0.0	52.5	2.6	52.3	109.1	4.8	107.6	149.1	5.7	146.3
75%	0.0	0.0	0.0	0.0	0.0	0.0	43.2	2.2	43.8	101.3	4.4	100.7	142.8	5.5	139.4
20-40															
25%	0.0	0.0	0.0	0.0	0.0	0.0	61.6	2.8	61.3	117.3	4.7	114.5	155.2	5.6	153.0
50%	0.0	0.0	0.0	0.0	0.0	0.0	48.6	2.2	49.3	107.8	4.1	107.0	149.2	5.2	147.1
75%	0.0	0.0	0.0	0.0	0.0	0.0	36.4	1.6	37.3	98.1	3.7	98.3	142.2	4.8	141.6
40-60															
25%	0.0	0.0	0.0	0.0	0.0	0.0	60.5	2.6	60.1	113.5	4.1	112.3	150.7	4.9	148.8
50%	0.0	0.0	0.0	0.0	0.0	0.0	47.6	1.8	48.0	101.7	3.2	100.8	139.1	4.1	138.4
75%	0.0	0.0	0.0	0.0	0.0	0.0	33.4	1.2	34.3	88.6	2.4	88.9	127.6	3.0	127.7
60+															
25%	0.0	0.0	0.0	0.0	0.0	0.0	53.2	3.3	52.3	92.4	4.9	89.6	119.3	5.8	115.9
50%	0.0	0.0	0.0	0.0	0.0	0.0	39.4	3.1	37.9	66.4	4.8	63.0	84.8	5.7	79.8
75%	0.0	0.0	0.0	0.0	0.0	0.0	29.6	3.0	27.0	46.8	4.6	42.5	56.9	5.6	51.9

Table S11. Deaths (in thousands) for a second-wave scenario in New York City. Each major row heading denotes the age group which adopts salutary sheltering, and the sub-headings denote the fraction of the group which shelters. The major column headings give the level of contact amongst individuals who do not shelter. The entry "Total" gives the median number of total deaths (in thousands) in each scenario, while "0-59" and "60+" give the median number of total deaths in each segment of the population (under or over 60 years of age).

	5% contact			25% contact			50% contact			75% contact			100% contact		
	Total	0-59	60+	Total	0-59	60+	Total	0-59	60+	Total	0-59	60+	Total	0-59	60+
0-19															
25%	0.0	0.0	0.0	0.3	0.0	0.0	31.0	6.0	24.0	63.6	11.0	51.4	83.5	13.5	70.0
50%	0.0	0.0	0.0	0.0	0.0	0.0	25.4	4.9	19.5	59.4	10.6	47.9	80.4	13.3	67.0
75%	0.0	0.0	0.0	0.0	0.0	0.0	19.9	4.1	17.3	55.7	10.2	44.6	78.1	13.0	63.7
20-40															
25%	0.0	0.0	0.0	0.1	0.0	0.0	26.5	4.9	19.6	59.4	9.7	48.6	80.5	12.7	67.4
50%	0.0	0.0	0.0	0.0	0.0	0.0	16.8	2.9	14.9	49.6	8.4	41.3	73.1	11.2	61.4
75%	0.0	0.0	0.0	0.0	0.0	0.0	8.7	1.5	7.2	40.8	6.5	32.8	64.9	9.4	54.5
40-60															
25%	0.0	0.0	0.0	0.1	0.0	0.0	28.3	4.9	22.1	58.9	9.0	49.2	79.3	11.1	67.3
50%	0.0	0.0	0.0	0.0	0.0	0.0	21.1	3.1	16.8	50.5	6.7	42.6	71.3	8.6	62.0
75%	0.0	0.0	0.0	0.0	0.0	0.0	13.2	1.8	13.0	42.5	5.1	36.3	63.3	6.3	56.0
60+															
25%	0.0	0.0	0.0	0.7	0.0	0.0	29.0	6.8	20.8	53.6	11.2	40.6	68.7	13.6	54.2
50%	0.0	0.0	0.0	0.1	0.0	0.0	23.0	6.5	14.7	41.9	11.0	29.0	52.8	13.5	37.6
75%	0.0	0.0	0.0	0.0	0.0	0.0	17.4	6.2	9.9	31.0	10.6	18.9	38.9	13.6	23.2

Table S12. Deaths (in thousands) for a fully susceptible population in Hubei. Each major row heading denotes the age group which adopts salutary sheltering, and the sub-headings denote the fraction of the group which shelters. The major column headings give the level of contact amongst individuals who do not shelter. The entry "Total" gives the median number of total deaths (in thousands) in each scenario, while "0-59" and "60+" give the median number of total deaths in each segment of the population (under or over 60 years of age).

	5% contact			25% contact			50% contact			75% contact			100% contact		
	Total	0-59	60+	Total	0-59	60+	Total	0-59	60+	Total	0-59	60+	Total	0-59	60+
0-19															
25%	0.0	0.0	0.0	0.0	0.0	0.0	145.4	28.7	116.6	257.3	52.1	205.3	312.0	63.1	249.4
50%	0.0	0.0	0.0	0.0	0.0	0.0	65.8	13.5	51.8	245.3	50.3	195.7	303.3	61.8	241.7
75%	0.0	0.0	0.0	0.0	0.0	0.0	7.4	1.5	5.9	235.5	48.6	186.4	293.5	60.5	233.0
20-40															
25%	0.0	0.0	0.0	0.0	0.0	0.0	128.7	24.2	104.9	242.5	47.1	195.5	299.0	58.7	240.7
50%	0.0	0.0	0.0	0.0	0.0	0.0	29.8	5.2	24.6	214.0	39.7	174.3	274.8	51.6	223.2
75%	0.0	0.0	0.0	0.0	0.0	0.0	0.3	0.0	0.2	177.5	31.1	146.6	248.8	44.9	204.2
40-60															
25%	0.0	0.0	0.0	0.0	0.0	0.0	137.3	24.3	113.1	241.3	43.8	197.6	293.9	53.0	241.4
50%	0.0	0.0	0.0	0.0	0.0	0.0	68.8	10.7	58.1	214.0	34.0	180.1	268.1	42.4	225.6
75%	0.0	0.0	0.0	0.0	0.0	0.0	4.2	0.6	3.6	184.4	25.3	159.0	240.1	32.3	207.9
60+															
25%	0.0	0.0	0.0	0.0	0.0	0.0	149.2	31.7	117.4	235.0	52.0	183.1	278.9	62.2	216.6
50%	0.0	0.0	0.0	0.0	0.0	0.0	126.9	29.1	97.7	208.0	50.1	157.7	244.0	60.5	183.6
75%	0.0	0.0	0.0	0.0	0.0	0.0	108.4	26.3	82.1	185.3	48.2	137.5	219.3	59.1	159.4

Table S13. Deaths (in thousands) for a fully susceptible population in Lombardy. Each major row heading denotes the age group which adopts salutary sheltering, and the sub-headings denote the fraction of the group which shelters. The major column headings give the level of contact amongst individuals who do not shelter. The entry "Total" gives the median number of total deaths (in thousands) in each scenario, while "0-59" and "60+" give the median number of total deaths in each segment of the population (under or over 60 years of age).

	5% contact			25% contact			50% contact			75% contact			100% contact		
	Total	0-59	60+	Total	0-59	60+	Total	0-59	60+	Total	0-59	60+	Total	0-59	60+
0-19															
25%	0.0	0.0	0.0	0.0	0.0	0.0	95.1	4.6	90.6	149.2	6.2	143.1	186.0	7.0	178.8
50%	0.0	0.0	0.0	0.0	0.0	0.0	85.0	4.1	81.0	141.7	5.9	135.6	179.5	6.9	172.6
75%	0.0	0.0	0.0	0.0	0.0	0.0	75.2	3.7	71.5	133.8	5.8	127.9	173.0	6.8	166.2
20-40															
25%	0.0	0.0	0.0	0.0	0.0	0.0	94.9	4.3	90.6	149.5	5.9	143.5	187.1	6.8	180.3
50%	0.0	0.0	0.0	0.0	0.0	0.0	83.3	3.6	79.7	141.8	5.4	136.4	181.8	6.4	175.3
75%	0.0	0.0	0.0	0.0	0.0	0.0	71.1	2.9	68.1	133.1	4.9	128.3	175.5	6.0	169.7
40-60															
25%	0.0	0.0	0.0	0.0	0.0	0.0	93.5	3.9	89.6	145.5	5.3	140.2	181.6	6.1	175.5
50%	0.0	0.0	0.0	0.0	0.0	0.0	80.9	3.0	77.8	132.9	4.3	128.7	171.4	5.0	166.4
75%	0.0	0.0	0.0	0.0	0.0	0.0	66.8	2.2	64.6	121.2	3.4	117.7	159.8	3.9	155.9
60+															
25%	0.0	0.0	0.0	0.0	0.0	0.0	80.4	4.7	75.6	118.9	6.2	112.6	145.8	7.0	138.7
50%	0.0	0.0	0.0	0.0	0.0	0.0	60.6	4.5	56.2	87.2	6.0	81.2	105.4	6.9	98.4
75%	0.0	0.0	0.0	0.0	0.0	0.0	45.6	4.4	41.1	62.5	5.9	56.6	72.4	6.7	65.6

Table S14. Deaths (in thousands) for a fully susceptible population in New York City. Each major row heading denotes the age group which adopts salutary sheltering, and the sub-headings denote the fraction of the group which shelters. The major column headings give the level of contact amongst individuals who do not shelter. The entry "Total" gives the median number of total deaths (in thousands) in each scenario, while "0-59" and "60+" give the median number of total deaths in each segment of the population (under or over 60 years of age).

	5% contact			25% contact			50% contact			75% contact			100% contact		
	Total	0-59	60+	Total	0-59	60+	Total	0-59	60+	Total	0-59	60+	Total	0-59	60+
0-19															
25%	0.0	0.0	0.0	19.1	4.5	14.4	72.2	14.7	57.0	100.4	18.7	81.7	118.5	20.6	97.9
50%	0.0	0.0	0.0	9.2	2.2	7.0	66.5	14.1	52.6	96.7	18.3	78.5	114.9	20.3	94.6
75%	0.0	0.0	0.0	0.0	0.0	0.0	59.9	13.0	47.5	93.1	18.1	75.0	111.8	20.1	91.7
20-40															
25%	0.0	0.0	0.0	20.3	4.4	15.9	69.6	13.7	55.7	98.9	17.8	81.1	115.9	19.5	96.4
50%	0.0	0.0	0.0	13.6	3.1	10.6	60.7	12.0	48.7	91.3	16.0	75.3	111.0	18.4	92.6
75%	0.0	0.0	0.0	8.7	1.9	6.7	52.7	9.9	42.6	83.4	14.3	69.1	104.9	17.0	87.8
40-60															
25%	0.0	0.0	0.0	20.8	4.2	16.6	69.1	13.0	56.2	96.5	16.0	80.5	114.2	17.8	96.4
50%	0.0	0.0	0.0	16.5	3.1	13.0	60.8	10.3	50.5	88.6	13.1	75.5	107.0	14.6	92.4
75%	0.0	0.0	0.0	11.2	2.2	9.1	52.9	8.0	44.7	80.0	10.4	69.6	99.0	11.6	87.4
60+															
25%	0.0	0.0	0.0	20.9	5.6	15.7	63.7	15.3	48.1	85.3	18.7	66.6	98.5	20.4	78.2
50%	0.0	0.0	0.0	17.4	5.4	12.2	51.1	15.0	36.0	67.8	18.7	49.2	78.2	20.5	57.7
75%	0.0	0.0	0.0	14.0	5.1	9.0	40.5	15.0	25.5	51.5	18.3	33.3	58.5	20.4	38.1

References

1. P Van den Driessche, M Li, J Muldowney, Global stability of SEIRS models in epidemiology. *Can. Appl. Math. Q.* **7**, 409–425 (1999).
2. F Ball, E Knock, P O'Neill, Stochastic epidemic models featuring contact tracing with delays. *Math. Biosci.* **266**, 23–35 (2015).
3. G Roth, et al., Global, regional, and national age-sex-specific mortality for 282 causes of death in 195 countries and territories, 1980–2017: A systematic analysis for the Global Burden of Disease Study 2017. *The Lancet* **392**, 1736–1788 (2018).
4. F Zhou, et al., Clinical course and risk factors for mortality of adult inpatients with COVID-19 in Wuhan, China: a retrospective cohort study. *The Lancet* (2020).
5. Y Bai, et al., Presumed asymptomatic carrier transmission of COVID-19. *The J. Am. Med. Assoc.* (2020).
6. C Rothe, et al., Transmission of 2019-nCoV infection from an asymptomatic contact in Germany. *New Engl. J. Medicine* (2020).
7. Z Du, et al., Serial interval of COVID-19 among publicly reported confirmed cases. *Emerg. Infect. Dis.* (2020).
8. R Li, et al., Substantial undocumented infection facilitates the rapid dissemination of novel coronavirus (SARS-CoV2). *Science* (2020).
9. K Prem, A Cook, M Jit, Projecting social contact matrices in 152 countries using contact surveys and demographic data. *PLoS Comput. Biol.* **13**, e1005697 (2017).
10. P Allison, *Survival analysis using SAS: a practical guide.* (SAS Institute), (2010).
11. D Collett, *Modelling survival data in medical research.* (CRC Press), (2015).
12. S Lauer, et al., The incubation period of coronavirus disease 2019 (COVID-19) from publicly reported confirmed cases: Estimation and application. *Annals Intern. Medicine* (2020).
13. Y Liu, R Eggo, A Kucharski, Secondary attack rate and superspreading events for SARS-CoV-2. *The Lancet* (2020).
14. J Zhang, et al., Changes in contact patterns shape the dynamics of the covid-19 outbreak in china. *Science* (2020).
15. Z Hu, X Peng, Household changes in contemporary China: An analysis based on the four recent censuses. *The J. Chin. Sociol.* **2**, 9 (2015).
16. D He, X Zhang, Z Wang, Y Jiang, China fertility report, 2006–2016. *China Popul. Dev. Stud.* **2**, 430–439 (2019).
17. United Nations, World population prospects 2019 (2019) https://population.un.org/wpp/.
18. Statista, Household structures in Italy in 2018 (2018) https://www.statista.com/statistics/730604/family-structures-italy/, Last Accessed: 2020-03-28.
19. Statista, Number of single-person households in Italy from 2012 to 2018 (2018) https://www.statista.com/statistics/728061/number-of-single-person-households-italy/, Last Accessed: 2020-03-28.
20. Statista, Number of couples with children in Italy from 2012 to 2018, by number of children (2018) https://www.statista.com/statistics/570106/number-of-couples-with-children-italy/, Last Accessed: 2020-03-28.
21. Statista, Biennial average number of household members in Italy from 2012 to 2018 (2018) https://www.statista.com/statistics/671945/biennial-average-number-of-families-with-children-italy/, Last Accessed: 2020-03-28.
22. Statista, Number of single parents in Italy from 2011 to 2018, by number of children (2018) https://www.statista.com/statistics/570234/number-of-single-parents-with-children-in-italy-by-number-of-children/, Last Accessed: 2020-03-28.
23. E Carrà, M Lanz, S Tagliabue, Transition to adulthood in Italy: An intergenerational perspective. *J. Comp. Fam. Stud.* **45**, 235–248 (2014).
24. MP Center, Integrated public use microdata series, international: Version 7.2 [dataset] (2019) https://doi.org/10.18128/D020.V7.2.
25. Y Xu, et al., Prevalence and Control of Diabetes in Chinese Adults. *The J. Am. Med. Assoc.* **310**, 948–959 (2013).
26. Z Wang, et al., Status of hypertension in China. *Circulation* **137**, 2344–2356 (2018).
27. P Modesti, et al., Prevalence, awareness, treatment, and control of hypertension among Chinese first-generation migrants and Italians in Prato, Italy: The CHIP study. *Int. J. Hypertens.* (2017).
28. C for Disease Control, Prevention, New York City diabetes ABC profile 2011–2012 (2017).
29. NYCD of Health, M Hygiene, Hypertension in New York City: Disparities in prevalence (2016).
30. Y Tatsumi, T Ohkubo, Hypertension with diabetes mellitus: significance from an epidemiological perspective for Japanese. *Hypertens. Res.* **40**, 795–806 (2017).
31. R Verity, et al., Estimates of the severity of COVID-19 disease. *medRxiv* (2020).
32. CCR Team, Severe outcomes among patients with coronavirus disease 2019 (COVID-19)—United States, February 12–March 16, 2020 (2020) https://www.cdc.gov/mmwr/volumes/69/wr/mm6912e2.htm.
33. CC for Disease Control, Prevention, The epidemiological characteristics of an outbreak of 2019 novel coronavirus diseases (COVID-19). *China CDC Wkly.* **2**, 113–122 (2020) http://weekly.chinacdc.cn//article/id/e53946e2-c6c4-41e9-9a9b-fea8db1a8f51.
34. X Peng, China's demographic history and future challenges. *Science* **333**, 581–587 (2011).
35. South China Morning Post, Coronavirus: China's first confirmed COVID-19 case traced back to November 17 (2020) https://www.scmp.com/news/china/society/article/3074991/coronavirus-chinas-first-confirmed-covid-19-case-traced-back.
36. Italian National Institute of Statistics, Median age in Lombardy (2019) https://www4.istat.it/it/lombardia/dati?qt=gettable&dataset=DCIS_INDDEMOG1&dim=21,0,0, Last Accessed: 2020-03-28.

37. CW Factbook, Field listing: median age (2020) https://www.cia.gov/library/publications/the-world-factbook/fields/343.html, Last Accessed: 2020-03-28.
38. F Carinci, COVID-19: Preparedness, decentralisation, and the hunt for patient zero. *Br. Med. J.* (2020).
39. ND of Health, M Hygiene, Coronavirus disease 2019 (covid-19) daily data summary (2020) https://www1.nyc.gov/assets/doh/downloads/pdf/imm/covid-19-daily-data-summary-deaths-05172020-1.pdf.
40. Politico, Italian doctors on coronavirus frontline face tough calls on whom to save (2020) https://www.politico.eu/article/coronavirus-italy-doctors-tough-calls-survival/.
41. Y Mounk, The extraordinary decisions facing Italian doctors (2020) https://www.theatlantic.com/ideas/archive/2020/03/who-gets-hospital-bed/607807/.
42. Google, COVID-19 community mobility reports (2020) https://www.google.com/covid19/mobility/.
43. Unacast, Social distancing scoreboard (2020) https://www.unacast.com/covid19/social-distancing-scoreboard.
44. AS Gonzalez-Reiche, et al., Introductions and early spread of sars-cov-2 in the new york city area. *medRxiv* (2020).
45. B Carey, J Glanz, Hidden outbreaks spread through U.S. cities far earlier than americans knew, estimates say. *The New York Times* (2020) https://www.nytimes.com/2020/04/23/us/coronavirus-early-outbreaks-cities.html.
46. U.S. Bureau of Labor Statistics, American time use survey (2018) https://www.bls.gov/tus/.
47. N Qualls, et al., Community mitigation guidelines to prevent pandemic influenza—united states, 2017. *CDC MMWR Recommendations and Reports* **66**, 1 (2017).
48. Oklahoma County Department of Health, Social distancing fact sheet (2019) https://www.occhd.org/application/files/3715/7013/9751/Social_Distancing.pdf, Last Accessed: 2020-04-05.
49. F Ahmed, N Zviedrite, A Uzicanin, Effectiveness of workplace social distancing measures in reducing influenza transmission: a systematic review. *BMC Public Heal.* **18**, 518 (2018).
50. P Totterdell, Work schedules in *Handbook of work stress*, eds. J Barling, EK Kelloway, MR Frone. (Sage publications), (2004).
51. L DeRigne, P Stoddard-Dare, L Quinn, Workers without paid sick leave less likely to take time off for illness or injury compared to those with paid sick leave. *Heal. Aff.* **35**, 520–527 (2016).
52. MW Fong, et al., Nonpharmaceutical measures for pandemic influenza in nonhealthcare settings—social distancing measures. *Emerg. Infect. Dis.* **26** (2020).
53. F Aimone, The 1918 influenza epidemic in new york city: a review of the public health response. *Public Heal. Reports* **125**, 71–79 (2010).
54. L Ecola, T Light, Equity and congestion pricing. *RAND Corp.*, 1–45 (2009).
55. NBC News, Walmart will limit customers and create one-way traffic inside its stores (2020) https://www.nbcnews.com/news/us-news/walmart-will-limit-customers-create-one-way-traffic-inside-its-n1176461.
56. NBC Connecticut, New social distancing measures take effect at stores across Connecticut (2020) https://www.nbcconnecticut.com/news/local/new-social-distancing-measures-enacted-at-stores-across-connecticut/2249937/.
57. A Selyukh, Supermarkets add 'senior hours' for vulnerable shoppers (2020) https://www.npr.org/sections/coronavirus-live-updates/2020/03/19/818488098/supermarkets-add-senior-hours-for-vulnerable-shoppers.
58. Centers for Disease Control and Prevention, Interim pre-pandemic planning guidance: community strategy for pandemic influenza mitigation in the united states (2007).
59. WGNTV, This student created a network of 'shopping angels' to help the elderly get groceries during the COVID-19 pandemic (2020) https://wgntv.com/news/this-student-created-a-network-of-shopping-angels-to-help-the-elderly-get-groceries-during-the-covid-19-pandemic/.
60. American Broadcasting Company, Santa Monica volunteers protect seniors with new grocery delivery service (2020) https://abc7.com/coronavirus-seniors-covid-19-groceries/6045815/.
61. K Gardner, K Lister, 2017 state of telecommuting in the U.S. employee workforce (2017) https://www.flexjobs.com/2017-State-of-Telecommuting-US/.
62. T Noah, 'it makes me very angry': Coronavirus damage ripples across the workforce (2020) https://www.politico.com/news/2020/03/09/america-workers-outbreak-uncertainty-124191.
63. Drew Desilver, As coronavirus spreads, which U.S. workers have paid sick leave – and which don't? (2020) https://www.pewresearch.org/fact-tank/2020/03/12/as-coronavirus-spreads-which-u-s-workers-have-paid-sick-leave-and-which-dont/.
64. D Card, Labor supply with a minimum hours threshold in *Carnegie-Rochester Conference Series on Public Policy*. (Elsevier), Vol. 33, pp. 137–168 (1990).
65. C for Disease Control, Prevention, Interim pre-pandemic planning guidance: Community strategy for pandemic influenza mitigation in the united states— early, targeted, layered use of nonpharmaceutical interventions (2007) https://www.cdc.gov/flu/pandemic-resources/pdf/community_mitigation-sm.pdf.
66. A Handel, IM Longini Jr, R Antia, What is the best control strategy for multiple infectious disease outbreaks? *Proc. Royal Soc. B: Biol. Sci.* **274**, 833–837 (2007).
67. WHO China, Report of the WHO-China Joint Mission on Coronavirus Disease 2019 (COVID-19) (2020) https://www.who.int/docs/default-source/coronaviruse/who-china-joint-mission-on-covid-19-final-report.pdf.
68. Q Li, et al., Early transmission dynamics in Wuhan, China, of novel coronavirus–infected pneumonia. *New Engl. J. Medicine* (2020).
